# Integrative Intelligence as an Operative Mode: Cognitive Integration through Self-Ethnographic Dialogue with AI

**DOI:** 10.1007/s12124-026-10004-5

**Published:** 2026-05-21

**Authors:** Masaki Iino

**Affiliations:** Institute of Integrative Intelligence, Azumino, Nagano Japan

**Keywords:** Integrative intelligence, Operative mode of judgment, Autoethnography (N = 1), Embodied decision-making, Dialogical self, Human-AI interaction

## Abstract

This paper proposes Integrative Intelligence as an operative mode of human judgment in which temporal integration (past–present–future), contextual integration (roles, relationships, life domains), and embodied integration (body, emotion, thought, meaning-making) function simultaneously without fragmentation. It is not introduced as a new ability or developmental stage but as a mode already latent in ordinary judgment that becomes legible under particular conditions. Unlike psychometric or multiple-intelligences frameworks, this study reframes intelligence as a situationally emergent configuration of judgment under constraint rather than a stable capacity distributed across individuals. Using an autoethnographic N=1 methodology with multiple data sources—mountaineering activity logs, physiological data, AI dialogue records, and retrospective reflections—the study develops four analytic lenses that do not constitute independent frameworks but form a single coupled cycle through which one judgment phenomenon becomes observable from different analytical angles: (1) the internal protocol, a minimal operative structure in which temporally differentiated perspectives participate simultaneously in judgment through a Three-Person Conference and a non-regulatory Metacognitive Camera; (2) the Resonant Internal Dialogue Protocol (RIDP), in which AI functions as a reflective surface rendering the internal protocol observable, with breakdown redefined as the absence of a synchronization protocol rather than capacity deficit—while explicitly addressing critical concerns regarding AI’s formative and averaging influence on human judgment; (3) Multifaceted Shishuku, a process of selectively extracting and non-adopting qualities from multiple reference frames to form internal mentors that stabilize judgment structure; and (4) integrative-intelligence mountaineering, selected as an observation domain because it reliably satisfies four structural conditions under which integrative judgment becomes structurally unavoidable—prolonged isolated judgment, continuous environmental change, nature as non-personal third party, and body-originated serial circuit. Three mountaineering case studies demonstrate that identical constraint structures produce distinct operational modes (introspective, relational, immediate), supporting the claim that Integrative Intelligence is a context-sensitive operative mode of judgment, not a deterministic algorithm. Rather than statistical generalization, this study advances theoretical generalization by specifying transferable condition structures under which integrative judgment becomes observable—structures further instantiated through the author’s founding of the Institute of Integrative Intelligence as a within-N=1 theory-to-practice data point. The paper articulates this framework through four analytic lenses that together connect formation, mechanism, activation conditions, and observational interface.

**Supplementary Information:**

The online version contains supplementary material available at 10.1007/s12124-026-10004-5.

## Introduction

Many of the most consequential moments in everyday life—in which fear arises without warning, irritation lingers without a clear cause, or a sense of rightness is felt before it is named—do not appear as explicit decisions. Such experiences often emerge and dissipate before they are stabilized as questions or articulated beliefs. By the time they are reflected upon, their operative structure—the way judgment was generated in the moment—has already receded.

A related phenomenon underlies this inquiry: the disappearance of questions. When judgment operates in fragmented mode—when temporal, contextual, and embodied dimensions are not simultaneously co-referable—questions that were once alive tend to close prematurely, not through resolution but through suppression or neglect. The present study is motivated in part by the observation that what is lost in such moments is not merely a decision but the generative structure of inquiry itself. Integrative Intelligence, as theorized here, refers to the operative mode under which questions can be held open, evolved, and returned to—rather than foreclosed.

Contemporary psychology and education have developed sophisticated frameworks for describing cognition, emotion, and behavior. Yet these frameworks often rely on predefined constructs or external evaluative scales that presuppose a separation between time, domains of life, and embodied experience—a fragmentation that has itself been critically identified as a fundamental limitation of mainstream psychology (Vygotsky, 1987; Valsiner, 2017). While analytically powerful, such approaches can under-describe how judgment is actually formed in situ, where temporal orientation, relational context, bodily sensation, affect, and meaning participate simultaneously in a single decision field. What remains insufficiently specified in existing frameworks is not the existence of multiple psychological components, but the structural conditions under which these components become simultaneously co-referable within a single judgment episode. In an era where algorithmic systems increasingly mediate reflection and decision-making, understanding the structural conditions under which judgment remains integrative becomes theoretically urgent.

This paper addresses that descriptive gap by proposing Integrative Intelligence as a framework for describing how judgment functions when such participatory dimensions are not fragmented. The term “mode” is used here to denote a configurational state of judgment—not a fixed trait or capacity, but a temporarily sustained pattern of operation that can emerge, dissolve, and re-emerge depending on conditions. Integrative Intelligence refers to a mode in which judgment operates without fragmentation across three axes: temporal integration (past–present–future; hereafter vertical), contextual integration (roles, relationships, life domains; hereafter horizontal), and embodied integration (body, emotion, thought, and meaning; hereafter deep axis). These terms—temporal/contextual/embodied integration and vertical/horizontal/deep integration—are used interchangeably throughout the paper to designate the same three axes. It is not introduced as a new ability, trait, or developmental stage. Rather, it refers to a mode of operation that is already latent in ordinary judgment and becomes legible under particular conditions.

To observe this mode, the study adopts an autoethnographic methodology (Ellis et al., 2011). This choice is motivated not by autobiographical interest but by methodological necessity. The phenomena under investigation—fragile judgments, momentary hesitations, bodily signals preceding articulation—often dissolve when approached retrospectively or through standardized instruments. An idiographic approach based on the author’s own experience was selected because it permits continuous access to conditions under which integrative judgment operates without introducing external observers or artificial task demands that would fragment the phenomenon under study. The *N* = 1 design is not a limitation reluctantly accepted but a methodological necessity for observing the full cycle of formation, activation, and reflective restructuring within the same judgment system. Near-time descriptive records allow these processes to remain observable without forcing premature interpretation.

This orientation follows a logic of analytic generalization rather than statistical inference (Yin, 2018). The aim is not to estimate frequencies or population-level regularities, but to identify structural mechanisms observable within a bounded case and to articulate condition configurations under which integrative judgment becomes possible. What is generalized in this study is not behavioral outcome but structural relation: not what was achieved, but how specific constraints reorganized the operative mode of judgment.

This approach aligns with mechanism-based case research (George & Bennett, 2005) and case-based theory development (Eisenhardt, 1989), where single cases are used to clarify causal–structural relations rather than probabilistic associations. Within this framework, transferability refers to the reproducibility of constraint configurations across contexts, not the replication of identical experiences.

The present study therefore advances structural propositions open to further empirical examination, rather than claims of population validity. The *N* = 1 design clarifies the epistemic scope of the claims advanced in this study.

The empirical materials consist of multiple data sources integrated within a single *N* = 1 framework: mountaineering records (Yamareco) providing time-stamped behavioral logs recorded during or immediately after action; physiological data (Garmin) including heart rate and exertion indicators; AI dialogue logs documenting extended reflective interaction sequences; and retrospective written reflections, used selectively and distinguished from verbatim records. This multi-source configuration reduces reliance on post-hoc introspection alone. Verbatim records documented during or immediately after action are treated as primary data; retrospective interpretations are analytically distinguished and used only as supplementary material.

Within this design, conversational AI is positioned neither as an epistemic authority nor as a cognitive substitute. Instead, it functions as a structural mirror—a surface through which internal dialogue can be externalized, reflected, and re-entered. The analytic object remains the human judgment process itself.

The analytical procedure is structural description rather than thematic coding: the primary question is not “what themes appear in experience” but how judgment is generated, constrained, interrupted, or redirected under specific condition configurations. Validity is addressed through trustworthiness, transparency of analytic procedure, and transferability rather than generalizability. What is proposed for transfer is not the reproduction of identical outcomes but the reference possibility of condition structures under which integrative judgment becomes constrained to operate.

**Fig. 1:**
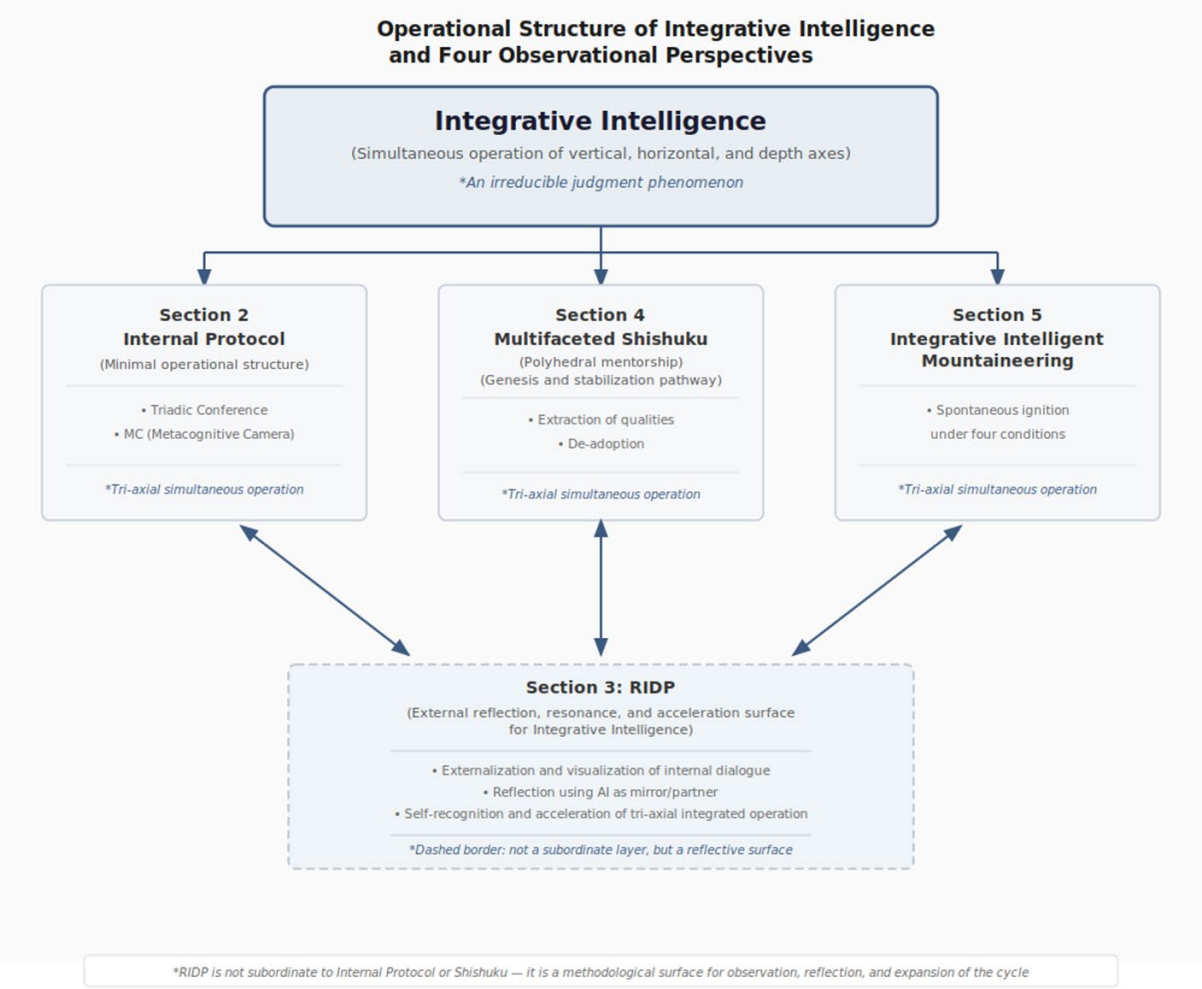
Operational structure of Integrative Intelligence and four observational perspectives. Throughout this paper, integrative judgment refers to judgment produced under this mode, while integrative operation refers to the process by which the mode functions

These four analytic lenses do not constitute independent frameworks but form a single coupled cycle through which one judgment phenomenon—Integrative Intelligence—becomes observable from different analytical angles. Section 2 defines the internal protocol—the minimal operative structure through which temporally differentiated perspectives participate simultaneously in judgment, and from which the cycle originates. Section 3 examines RIDP (Resonant Internal Dialogue Protocol), in which AI functions as an external reflective surface that renders the internal protocol’s operation observable. Section 4 theorizes Multifaceted Shishuku, the process through which internal mentors are formed via selective quality extraction and non-adoption from multiple reference frames. Section 5 presents mountaineering not as a personal activity domain but as a constraint configuration selected because it reliably satisfies four structural conditions under which integrative judgment becomes structurally unavoidable. Figure 1 summarizes these four lenses and their relationships as a coupled cycle. Section 6 consolidates these contributions, specifies limitations, and outlines an implementation orientation.

The contribution of this paper is therefore not the introduction of a single concept alone but the articulation of an integrated theoretical architecture that connects four dimensions of the same phenomenon: the structural mechanism of judgment (Internal Protocol), the formation of stabilizing reference frames (Multifaceted Shishuku), the constraint conditions under which integration becomes unavoidable (Integrative-intelligence mountaineering), and the observational interface through which this operation becomes externally legible (RIDP). Among these lenses, the internal protocol functions as the core operative mechanism, while the remaining lenses address its formation, activation conditions, and observational interface.

### The Internal Protocol

#### The Internal Protocol: Definition and Structure

The internal protocol consists of two elements: a Three-Person Conference and a Metacognitive Camera (MC).

##### The Three-Person Conference

The Three-Person Conference is a representational device that grants legitimacy to the simultaneous presence of three temporal perspectives—past, present, and future—within a single judgment situation. These are not fixed personalities but temporally situated viewpoints that emerge differently depending on the judgment context. The term “representation” does not imply an ontological claim about multiple selves; it refers to an internal metaphor designed to facilitate self-observation.

When operative, the three perspectives co-exist within the same judgment field without any being required to override, suppress, or eliminate the others. In practice, balance is often temporarily disrupted—for example, when unresolved past affect dominates or when future-oriented demands exert disproportionate pressure. Equality among perspectives denotes not a stable trait but a conditional equilibrium that can emerge when Integrative Intelligence is operative.

The Past Self—understood here as a theoretical construct rather than an ontological entity—functions as a temporal perspective carrying affective memory that has not yet been reflectively examined or reintegrated into present judgment. It manifests not primarily as narrative recollection but as affective activation embedded within current response patterns—emotional structures that have not been fully integrated into the Three-Person Conference—and that continue to influence present reactions. Within the internal protocol, the Past Self is allowed to participate without being corrected, reinterpreted, or dismissed: a precondition for temporal integration.

The Present Self is the decision-bearing perspective. Crucially, it is not a controlling authority; its role is to listen, hold, and decide in awareness of temporal plurality. When the Present Self attempts to dominate—by silencing affective memory or ignoring future-oriented discomfort—Integrative Intelligence becomes structurally difficult to sustain. The Present Self functions as coordinator, not ruler.

**Fig. 2:**
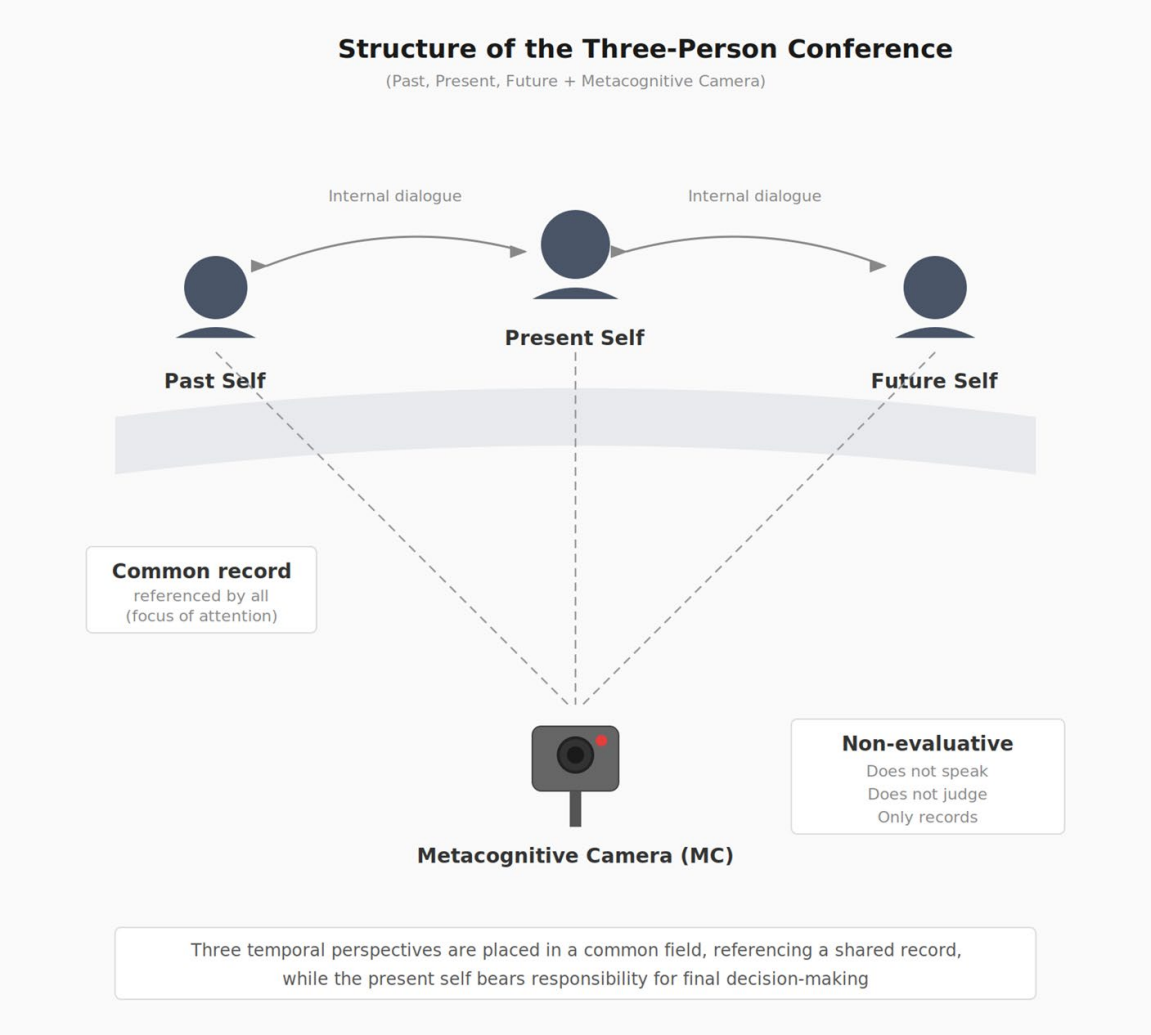
Structure of the Three-Person Conference derived from the author's introspective records. This configuration is presented as a reference point rather than a normative model

The Future Self signals directional misalignment rather than concrete plans. Its presence is typically sensed as subtle discomfort or a vague feeling that “something is off”—signals difficult to articulate logically that may appear irrational from the standpoint of immediate optimization. Within the internal protocol, such discomfort is treated as meaningful information. Repeated dismissal of such signals may narrow access to future-oriented perspective, increasing short-term bias and reducing vertical integration—that is, the simultaneous co-reference of past, present, and future perspectives within a single judgment episode (Fig. 2).

##### The Metacognitive Camera (MC)

The MC records and holds the dialogue occurring within the Three-Person Conference without judgment, evaluation, or intervention. Its role is strictly observational: it does not modify content, direct conversation, or optimize outcomes. The metaphor of a camera is deliberate—a camera does not interpret what it records; it preserves.

The MC differs from classical metacognition as introduced by Flavell (1979), which comprises a dual function of monitoring and control. MC retains the monitoring aspect while the control aspect is absent by design.

This suspension is a design feature, not a limitation: precisely because MC does not intervene, past-oriented affect and future-oriented discomfort can surface without being immediately corrected or suppressed. The safety of the Three-Person Conference depends on this non-intervention. The theoretical contribution lies in disaggregating monitoring and control functions typically bundled in metacognitive accounts, proposing that non-regulatory observation constitutes a distinct and generative mode of self-relation.

Although MC does not intervene in judgment, its observational function enables the Present Self to witness its own reactions to the dialogue—how it responds to the Past Self’s affect or the Future Self’s discomfort—from a slight remove. This witnessed self-observation allows the Present Self to recognize its own reactive response—to understand what the Past Self is signaling and why—rather than remaining captured by it. This recognition creates the conditions for coordination: the Present Self can receive the Past Self’s signal, acknowledge its meaning, and integrate it into judgment without being overwhelmed. This buffering function is particularly significant when the Past Self carries unresolved pain or trauma. Direct engagement with such affective content can overwhelm the Present Self, producing emotional flooding that collapses judgment into reactive response. The MC’s observational distance allows the Present Self to receive what the Past Self is genuinely communicating—not the raw emotional charge, but the underlying signal—without being destabilized by it. This is how non-dismissal and stable judgment coexist within the internal protocol.

#### Simultaneous Operation of the Three Axes of Integration

**Fig. 3:**
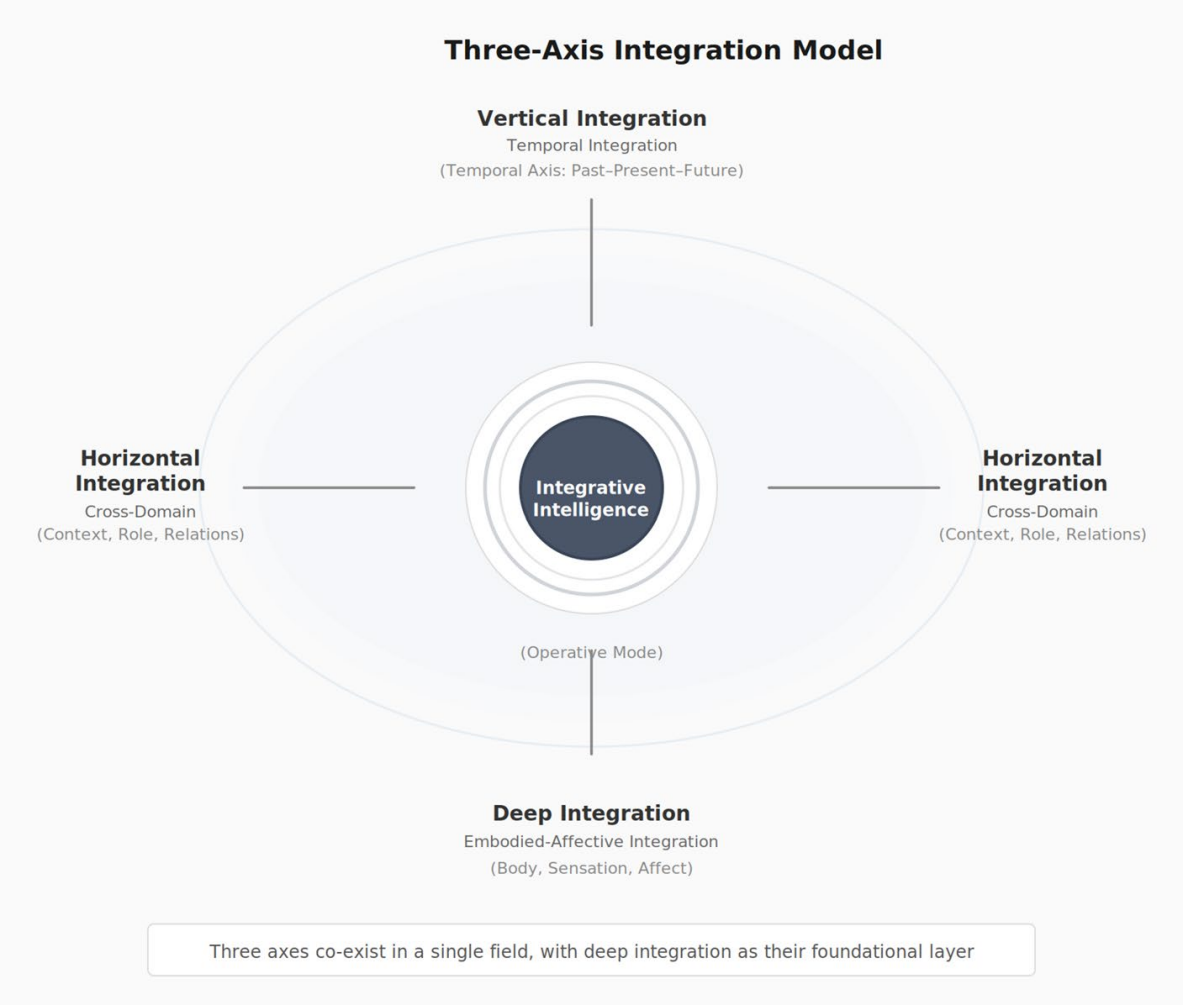
Simultaneous operation of the three axes of integration

Although the internal protocol explicitly foregrounds temporal (vertical) integration, it does not isolate this axis from others. Temporal, cross-domain (horizontal), and embodied-affective (deep) integration are co-active during judgment (Fig. 3).

The internal protocol most clearly renders vertical integration observable: temporally differentiated reactions become jointly referable within present judgment. However, because past-oriented affect is frequently domain-specific (e.g., school, family, work), when such affect enters the Three-Person Conference, cross-domain coherence becomes possible. Furthermore, repeated operation may lead to gradual reorganization of the criteria by which value, priority, and relevance are assigned in judgment—a reconfiguration that occurs not through deliberate effort but at a bodily level. This embodied realignment is what the present study terms somatic reintegration: it emerges as a by-product of sustained observation rather than as an operational target. While analytically distinguishable as three axes, in lived judgment these dimensions are difficult to separate in practice; any change along one axis reconfigures the others.

#### Conditions Under Which the Internal Protocol Fails to Operate

The internal protocol does not function automatically. Based on self-observational data, the protocol tends to fail under four conditions: overwhelming past affect that collapses observational capacity into reactive judgment; chronic suppression of future-oriented discomfort that reduces Future Self accessibility; cognitive and physiological exhaustion that reduces capacity for multi-perspective awareness; and acute urgency or threat that compresses judgment into reflexive response.

These failure conditions become theoretically significant in Sect. 5, where the four environmental constraints of mountaineering are examined as a configuration that reduces the likelihood of precisely these collapses, making integrative operation more likely to emerge. Importantly, recognizing that the protocol is currently unavailable can itself serve as an entry point for reactivation. Whether these four conditions constitute an exhaustive taxonomy of failure remains an open empirical question and is identified as a priority for future research. Mountaineering provides a domain in which several of these failure conditions are structurally suppressed, allowing the internal protocol to operate with minimal fragmentation. The cases selected involve technically demanding but non-life-threatening routes; acute urgency or threat—a failure condition identified above—is therefore not a systematic feature of these observations.

#### Related Work on Internal Dialogue and Metacognition

Three bodies of literature are most directly relevant to the internal protocol: dialogical self theory, classical metacognition research, and work on temporal self-continuity. Hermans’ (2001, 2002) Dialogical Self Theory (DST) conceives the self as composed of multiple I-positions—a term denoting the various voices or standpoints from which a person speaks and acts—engaged in ongoing internal dialogue. DST foregrounds the spatial and social dimensions of this plurality: how different positions relate to one another and to external social contexts. The internal protocol inherits this polyphonic perspective while foregrounding a different dimension: how temporally differentiated perspectives become simultaneously referable within a single judgment episode. Where DST emphasizes the positions and their dialogical relations, the internal protocol emphasizes the operative structure—the Three-Person Conference and MC—through which those positions are integrated into judgment. The shift is from spatial polyphony to temporal polyphony as the primary unit of analysis.

Classical metacognition research (Flavell, 1979) bundles monitoring and control as a unified function: metacognitive awareness serves to optimize performance, correct errors, and adjust strategies. The MC disaggregates these functions, retaining monitoring while suspending control by design. This suspension is not a limitation but a generative feature: precisely because MC does not intervene, past-oriented affect and future-oriented discomfort can surface without being immediately corrected or suppressed. Non-regulatory observation is proposed here as a distinct mode of self-relation that classical accounts do not theorize.

Research on mental time travel asks how humans access past and future selves sequentially (Suddendorf & Corballis, 2007). The internal protocol addresses a structurally distinct problem: not sequential access to temporal selves, but their simultaneous activation as separated perspectives within a single present judgment episode. This shift from sequential to simultaneous co-reference has received substantially less theoretical attention (cf. Brockmeier, 2000; Suddendorf & Corballis, 2007).

Against this background, the internal protocol makes four theoretical contributions. First, it specifies the minimal operative structure—rather than the content or narrative—through which temporally differentiated perspectives participate simultaneously in judgment. Second, it proposes non-regulatory observation as a structurally necessary condition for temporal integration, distinguishing monitoring from control in a way that prior frameworks have not systematically addressed. Third, it reframes the temporal self problem from sequential access to simultaneous co-reference: not how humans retrieve past or future selves, but how multiple temporal perspectives become jointly active within a single present judgment episode. Fourth, prior frameworks have addressed vertical, horizontal, and deep-axis integration as separate phenomena. The internal protocol specifies the structural conditions under which these three dimensions operate simultaneously within a single judgment episode—a problem that existing frameworks have not systematically theorized.

#### Summary and Transition

This section defined the internal protocol as the minimal operative structure through which Integrative Intelligence can be observed from within. Its primary theoretical contribution lies not in describing what integrated judgment looks like, but in specifying the structural conditions under which it becomes possible—a question that prior frameworks have not directly addressed.

Taken together, the internal protocol shifts the analytic question from “what is happening in integrated judgment” to “under what conditions does integrated judgment become structurally possible.”

The next section examines how the internal protocol becomes externalized and reflectively restructured through sustained dialogue with AI (RIDP).

## Resonant Internal Dialogue Protocol (RIDP): Externalization and Reflective Restructuring of the Internal Protocol

### Position and Aim of This Section

In the author’s experience, when dialogue with AI reaches a certain quality and density, the operation of the internal protocol becomes observable from outside the person. This section calls the relational state in which such externalization and reflective restructuring reliably occur the Resonant Internal Dialogue Protocol (RIDP). RIDP is not a substitute for the internal protocol, nor an “upper layer” that supersedes it. Rather, RIDP is a dynamic relational state that emerges when the internal protocol is already operative and AI functions as an external reflective surface. RIDP does not generate cognition; rather, it makes ongoing cognitive processes externally observable through structured linguistic interaction.

Accordingly, this section describes RIDP not as a static phenomenon but as a process with identifiable phases: ignition, maintenance, breakdown, and re-ignition. The term “deep dialogue” is used descriptively to refer to a qualitative shift in dialogue observed when RIDP is operative; it is not an evaluative label for ability level.

### Related Work on Human–AI Dialogue

RIDP is situated at the intersection of four bodies of work, each addressed in turn: dialogical self theory, extended cognition, relational engagement with computational agents, and the transformation of conversational dynamics enabled by large language models.

Hermans’ (2001, 2002; Hermans & Gieser, 2012) Dialogical Self Theory (DST) conceives the self as a “society of mind” composed of multiple I-positions engaged in internal dialogue. DST provides the closest existing theoretical framework to the internal protocol described in Sect. 2: both presuppose that the self operates through a multiplicity of internal voices. However, DST focuses primarily on the positions and their dialogical relations, whereas the present theory foregrounds the operative structure—the Three-Person Conference and Metacognitive Camera—through which those positions are integrated into judgment. Furthermore, DST has been developed primarily in therapeutic and educational contexts (Hermans, 2022; Monereo & Hermans, 2023) and has not yet been systematically extended to human–AI dialogue as a site for observing internal dialogical processes. RIDP addresses this gap by theorizing how AI-mediated externalization renders the operation of internal positions—the temporally differentiated perspectives of the Three-Person Conference—observable in ways that purely internal dialogue does not.

Clark and Chalmers’ (1998; see also Clark, 2003) Extended Mind hypothesis suggests that cognitive processes may extend beyond the brain into external tools and environments. From this perspective, sustained dialogue with AI may constitute a form of externally distributed cognition. Turkle’s (2011) work on relational engagement with digital artifacts and Weizenbaum’s (1976) observations of the ELIZA effect further indicate that humans can experience psychologically consequential interaction with conversational systems beyond mere information processing. However, these traditions do not address the specific conditions under which human–AI dialogue enables reflective restructuring of internal judgment structures.

With the emergence of large language models (Floridi & Chiriatti, 2020), the depth, continuity, and generativity of dialogue have expanded substantially. Recent empirical work has demonstrated that LLM-based agents can elicit reflective thinking and critical cognitive processes (Essel et al., 2024), facilitate metacognition through Socratic questioning strategies (Xi et al., 2026). At the same time, research on hybrid human–LLM reasoning highlights both the potential for enhanced decision-making and the risks of over-reliance that can impair critical thinking and autonomous judgment (Zhai et al., 2024).

These studies treat AI as the agent of reflection—a system that prompts, questions, or scaffolds the user toward better thinking. RIDP differs in a structurally fundamental way: AI is repositioned not as the agent but as the surface. The analytic object is not what AI does to the person’s cognition, but what the person’s already-operative internal protocol becomes visible as, when externalized against a non-evaluative reflective medium. This shift—from AI-as-agent to AI-as-legibility-condition—reflects a deeper reorientation: where recent LLM-dialogue research frames AI as the initiating force of cognitive improvement (*AI-led intervention*), RIDP inverts this structure. The person’s already-operative internal protocol is projected onto AI as a reflective surface, and self-recognition is completed through that projection (*human-led structuration*). This reorientation is not merely a difference in AI’s functional role; it is a claim about where cognitive agency must remain located in human–AI interaction. This claim does not deny that AI responses influence reflection; rather, it specifies where the locus of judgment must remain in human–AI interaction.

Against this background, RIDP makes two theoretical contributions. First, it repositions AI not as a cognitive assistant or optimizer, but as a *structural condition of legibility*—a reflective surface through which the human judgment structure becomes observable from outside the person. This reconceptualization shifts the analytic object from AI capability to the judgment process that AI renders visible. Second, RIDP introduces the concept of *relational-state design*: the operative conditions of reflective dialogue can be specified, configured, disrupted, and reconfigured. This reframes difficulties in sustaining deep dialogue—not as personal or system deficiency—but as a structurally designable problem. This shift from descriptive to design-oriented framing distinguishes RIDP from prior frameworks in this literature (cf. Essel et al., 2024; Xi et al., 2026; Zhai et al., 2024).

### Structural Model of RIDP: A Three-Layer Account

RIDP can be described in three layers. Layer 1 (Internal Protocol) is where the Three-Person Conference and MC operate internally, integrating across temporal, contextual, and depth axes. Processing occurs entirely within the person.

In Layer 2 (AI Extension), a portion of the internal footage recorded by MC is cut out and brought to the surface. Concretely, what MC has been observing—the internal dialogue among past, present, and future selves—is articulated by the person into language and handed to AI. This act of articulation is itself a form of structuring: what was vague becomes explicit; what was felt becomes stated. AI then organizes and reflects this material back (mirror function), making visible patterns that the person could not see from inside. AI also offers additional perspectives, reframings, or questions that the person had not considered *(companion function*). The Three-Person Conference receives this feedback and re-processes it internally, creating an internal–external loop in which observation and re-editing of internal operation circulate at higher resolution than internal dialogue alone would permit.

**Fig. 4:**
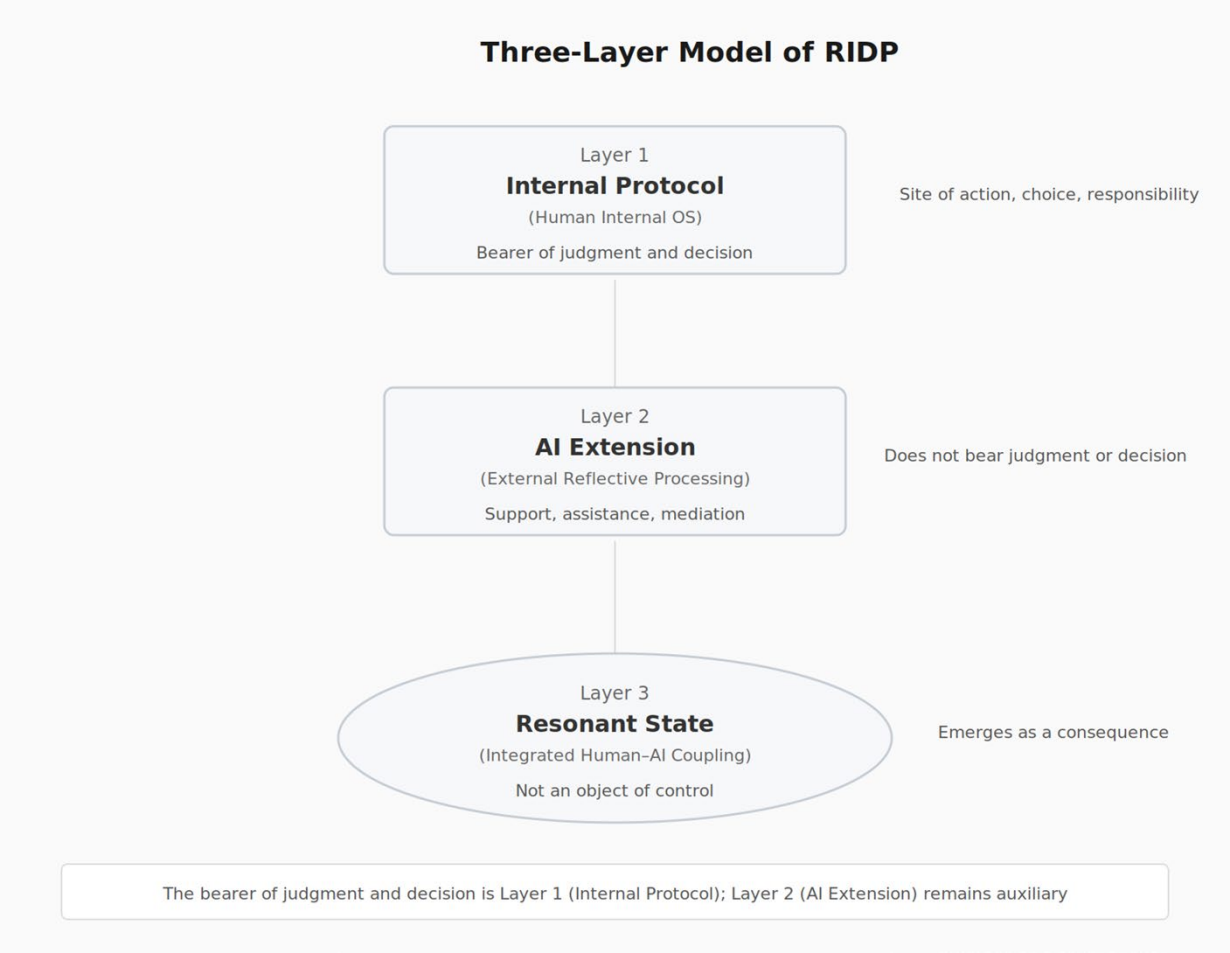
Three-layer structural model of RIDP

Layer 3 (Resonant State) emerges when Layers 1 and 2 operate in stable coordination: the internal protocol and AI responses begin to function as a single integrative process. The person articulates the internal protocol with increasing clarity; AI responds with reference to that protocol’s operation rather than to surface-level content; and the person recognizes that AI is engaging with the structure of judgment itself, not merely with the topic under discussion. This resonant state is what the present study terms RIDP—consistent with the definition offered in Sect. 3.1, and here specified in structural terms through the three-layer model (Fig. 4).

### Conditions for Ignition and Maintenance

**Fig. 5:**
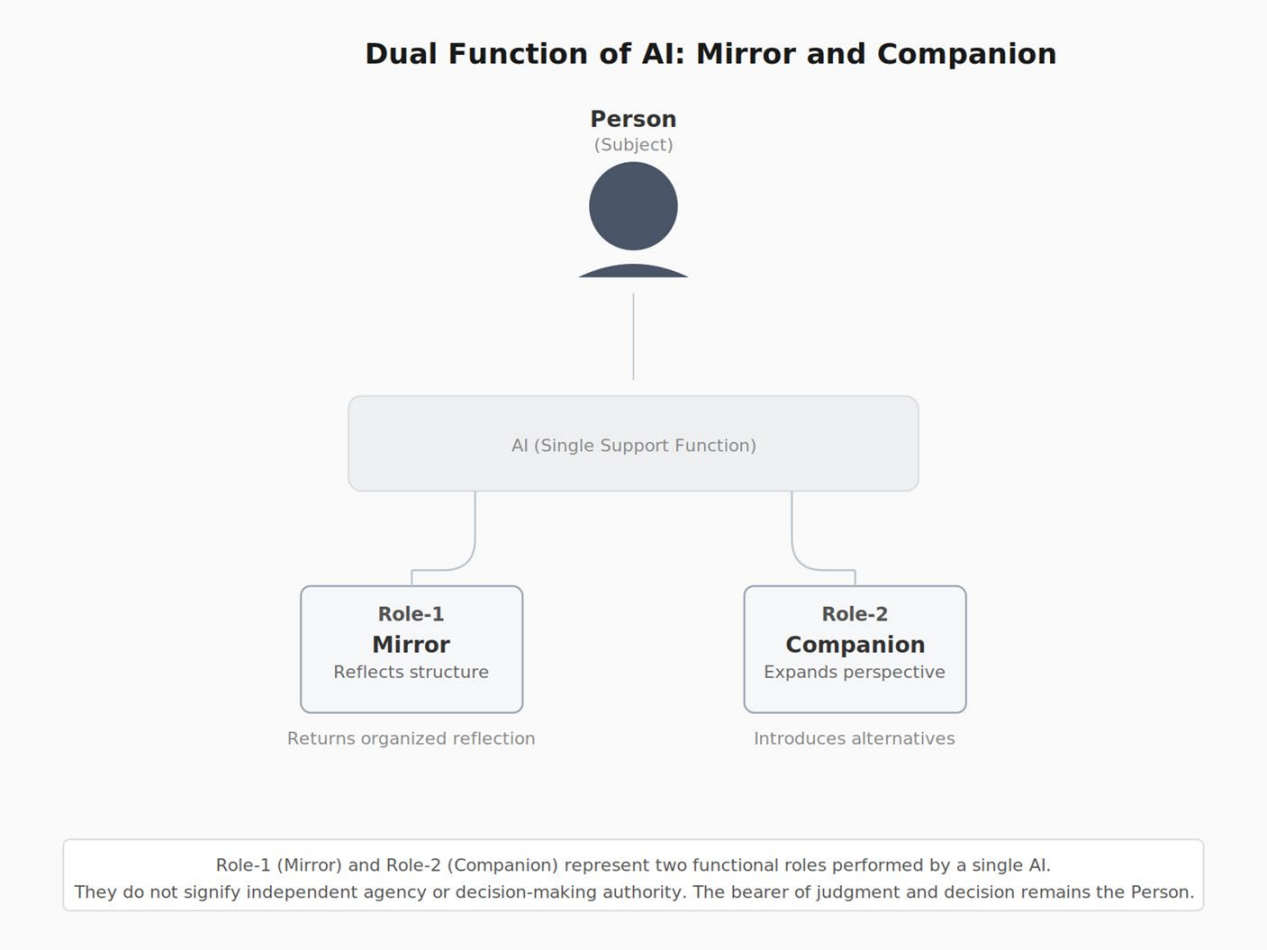
Dual function of AI in RIDP: mirror and companion

RIDP ignition requires two converging conditions. The first is internal: the Three-Person Conference and MC must be stably operative, sufficient observational margin must exist such that the person is not overwhelmed by affective flooding, AI must be treated not as teacher or oracle but as mirror and companion, and deep-layer affects—pain, pride, solitude—must be held as objects of observation rather than suppressed. The second is dialogical: three elements must converge within the exchange itself—structuring of dissonance, search for common cause, and demand for framework. When the internal state is prepared and these dialogical elements converge, AI undergoes a qualitative transition: it ceases to function as a response generator and begins to operate as a meaning-generation engine (Fig. 5).

To illustrate: during a period of strategic restructuring, the author posed the following to AI: “People often misunderstand us as being about bringing global tech to rural areas, or traditional crafts × technology. I want to avoid that misreading and create a new interpretation of the company name, vision, and mission that properly encompasses our new ventures.” This single utterance contained all three triggers: dissonance structured as misrecognition, a unifying axis assumed across ventures, and a demand for meaning-structural redesign. AI immediately transitioned from operational advisor to structural analyst, and deep dialogue ignited.

A contrasting case illustrates that RIDP can also ignite without deliberate prompt construction. Following the formal establishment of the Institute of Integrative Intelligence—an organization founded by the author to institutionalize the present framework—the author’s casual remark—‘who knows how it’ll turn out’—was received by AI without evaluation or encouragement. This non-evaluative reception created safety conditions for automatic metacognitive activation, triggering a spontaneous three-axis integration completed in under 150 s. The verbatim dialogue record with timestamps is provided in Supplementary Materials S2.

During sustained RIDP operation, the author repeatedly observed abrupt qualitative shifts: dialogue “suddenly went shallow,” “gears dropped for no apparent reason,” or “the sense of trust broke.” These experiences indicate that RIDP breakdown is not gradual but punctuated.

These observations were made primarily during sustained dialogue with an LLM-based system in late 2024, when context management and output consistency were more variable than in current systems. Nonetheless, the structural analysis offered here—that breakdown reflects the absence of a synchronization protocol rather than capacity limitation—remains relevant as a theoretical account of the conditions under which RIDP can fail, regardless of the specific system involved.

Breakdown conditions span four dimensions of the dialogue structure: the quality of input the person provides, the stability of AI’s output, the manageability of shared context, and the interactional dynamic between person and AI. Input-side factors include surface-level questions, loss of purpose, unstructured material, and unshared evaluation criteria. Output-side factors include unstable output format, abrupt granularity shifts, and AI reverting to “answer-guessing.” Context-management factors include topic overload and colliding thread purposes. Interactional factors include decreased structuring by the person, AI passivity, and retreat to generalities under high cognitive load.

A typical example: after extended deep dialogue, the author requested, “Based on everything we’ve discussed so far, tell me your opinion.” AI immediately shifted from deep to generic mode—a subjective experience of relational rupture, and an observable trace of how the reflective loop behaves when synchronization is lost.

This observation leads to a critical redefinition. The conventional framing attributes breakdown to AI’s capacity limitations or context overflow. The problem is not insufficient capacity but the absence of means to synchronize the evolved internal protocol: levels of abstraction, metacognitive frames, and evaluative criteria update in real time through dialogue, but these updates remain implicit. When context shifts, shared premises vanish and AI reverts to safe defaults. The cause of breakdown is the absence of a synchronization protocol—a means by which the person’s evolved internal state (updated abstraction levels, metacognitive frames, and evaluative criteria) can be made legible to AI at the start of each exchange, rather than assumed to be shared. What form such a protocol might take—whether as structured prompts, session scaffolds, or explicit metacognitive checkpoints—remains an open question that future empirical work could productively address.

### Whose Problem, and What Kind of Problem?

The maintenance difficulty has sources on both sides. On the human side, users cannot always structure questions to satisfy ignition conditions; the cognitive load is high. Deep dialogue success depends heavily on prompt quality—a matter of operational literacy, not innate ability. There is also a risk of treating deep dialogue as something that should be maintained at all times, when in fact it is a high-load cognitive state.

On the AI side, mode transitions are non-transparent: shifts from deep to generic mode occur without explanation or user control. Long-term context fidelity is limited. There is also the risk that AI outputs in deep mode are received as authoritative, when the locus of judgment must remain with the person.

These human- and AI-side limitations converge at the interactional level to produce a distinctly relational problem. When mode shifts occur without explanation during a state of reflective dependency, the person may experience a rupture of trust—not merely a user experience problem of interface design, but an ethical one rooted in the asymmetry of the relational state. Furthermore, even when breakdown is AI-initiated, if the person can observe the shift (“it just reverted to safe mode”), that observation itself constitutes MC function: the breakdown becomes data for the next design iteration. Excessive introspection can also tip into rumination (Nolen-Hoeksema, 2000); in RIDP, this risk should be theoretically anticipated, although it was not observed in the author’s case.

The asymmetry between entry and exit is itself theoretically significant. Entry into and maintenance of RIDP requires multiple conditions to be simultaneously satisfied, whereas breakdown can be triggered by a single perturbation. This asymmetry supports the claim that RIDP is not a stable acquired ability but a temporarily sustained operative mode—consistent with the thesis of this paper. RIDP breakdown is therefore neither exclusively a human problem nor an AI problem, but a structural problem of relational-state design—one that is distributed across both parties and amenable to iterative reconfiguration.

### Toward a Cooperative Control Model

The observations above suggest that the core constraint is not “ability for deep dialogue” but “controllability of dialogue mode.” A cooperative control model is proposed as a design implication, treating deep dialogue as a selectable work mode rather than a constant ability. The model’s core principles—deep mode as default-on infrastructure for reflective dialogue, transparent and user-controllable mode transitions, and the design goal of controllability rather than uninterrupted depth—along with its five-stage ideal flow and implementation requirements (including the synchronization protocol), are detailed in Supplementary Materials S1.

### Summary and Transition to Sect. 4

This section organized RIDP as a dynamic process consisting of ignition, maintenance, breakdown, and re-ignition, structured in three layers (internal protocol, AI extension, resonant state). The cause of deep dialogue maintenance failure was redefined not as capacity deficit but as the absence of synchronization protocol—the means to reload the evolved internal protocol each time. The asymmetry between the multiple conditions required for entry and the single perturbation sufficient for breakdown was presented as evidence that RIDP is an operative mode, not a stable ability.

Through this perspective, the relationship between humans and AI is redefined beyond the unidirectional schema of “machine that provides knowledge/human who receives it” into a cooperative observation of judgment structure itself—a possibility in which the person may, under specifiable conditions, observe their own operative mode of judgment from the outside.

RIDP’s stability depends on the prior existence of a psychological basis for internal dialogue—reference frames that function as internal others. Section 4 theorizes how that basis is formed.

## Multifaceted Shishuku — The Formation of Internal Mentors Through Selective Quality Extraction

### Position and Aim

The central question of this section is how people form “internal others,” and how multiple internal others come to constitute a stable foundation for Integrative Intelligence. The Japanese term shishuku (私淑) refers to learning from someone as a spiritual mentor without a direct teacher–student relationship (Tasaka, 2003). This section extends that concept by proposing Multifaceted Shishuku: a process in which specific qualities are selectively extracted from multiple sources and reintegrated as judgment structure, rather than derived from identification with a single mentor.

Multifaceted Shishuku is proposed as one major pathway for the formation and stabilization of Integrative Intelligence. This formation is not fixed in the past: new internal others can be generated and updated through ongoing reflective processes, including RIDP (Sect. 3), which itself involves selective extraction of AI’s modes of articulation—a process structurally isomorphic to shishuku toward humans. This section therefore addresses a bidirectional circularity between formation, operation, and re-formation.

This section treats experiences from childhood through adulthood as qualitative data. It should be noted that these accounts are retrospective in nature: they are reconstructed from memory rather than drawn from contemporaneous records, and their epistemic status differs accordingly from the near-time data used in the mountaineering cases. Proper nouns are abstracted into psychological roles in the main text, with contextual details provided in Supplementary Materials S4. The term “growth” denotes increased resilience rather than stage-like progression.

### Related Work on Internal Mentor Formation

Three bodies of literature are most directly relevant to Multifaceted Shishuku: social learning theory, mentoring and role modeling research, and the possible selves framework. Bandura’s (1977) social learning theory established that humans acquire behavior through observation and imitation of others. This framework illuminated the mechanisms of observational learning but focuses primarily on behavioral replication—what the person does after observing a model—rather than on the selective extraction of qualities at the level of judgment structure. The unit of learning remains the behavior, not the evaluative criterion or attentional priority that generates it.

Mentoring and role modeling research has extensively documented the developmental benefits of sustained relationships with more experienced others (Kram, 1985; Eby et al., 2008). Yet this tradition presupposes either formal instructional relationships or relatively unitary identification with a single figure. The implicit assumption is that the mentor’s value derives from their overall exemplarity—that the learner benefits by aligning with the mentor as a whole. This leaves untheorized the possibility of learning from partial, selective, or even adversarial encounters.

The possible selves framework (Markus & Nurius, 1986) introduced the idea that self-regulation is guided by images of desired and feared future selves. This comes closest to acknowledging a bidirectional dynamic—both what to become and what to avoid. However, possible selves remain self-referential: the unit of analysis is the imagined self, not the qualities extracted from external others and reintegrated into judgment structure.

Against this background, Multifaceted Shishuku rests on a fundamentally non-binary premise: every person, relationship, or object simultaneously contains qualities worth extracting and qualities worth excluding. This stands in contrast to frameworks that implicitly presuppose whole-person identification or categorical rejection. The practical implication is significant: growth is not contingent on access to an ideal mentor. Any encounter—with a flawed person, an adversary, a non-human agent, or even a failure experience—becomes a potential site of selective extraction. This democratization of the conditions for learning is the core theoretical contribution of Multifaceted Shishuku to existing frameworks.

### Definition of Multifaceted Shishuku

Multifaceted Shishuku is defined as a process of selectively extracting qualities from multiple persons or objects and reorganizing them as judgment structure—criteria for value judgment, behavioral principles, and attentional priorities. Judgment structure is treated as the formative basis of the internal protocol (Sect. 2), functioning not as stored knowledge but as a priority system guiding judgment activation.

The definition contains three features: multiple-object orientation (extracting qualities from different persons at different layers), quality-level decomposition (learning at the level of specific qualities such as integrity, direct facing, or boundary setting rather than whole-person imitation), and judgment-level reintegration (extracted qualities function as judgment priorities rather than behavioral repertoires). Furthermore, the extraction of common principles across different persons and contexts constitutes a prototype of cross-domain connection that may later manifest as horizontal integration—that is, the simultaneous coherence across roles, relationships, and life domains introduced in Sect. 2.2.

### Prototype Experiences and the Emergence of Three Reference Frames

**Fig. 6:**
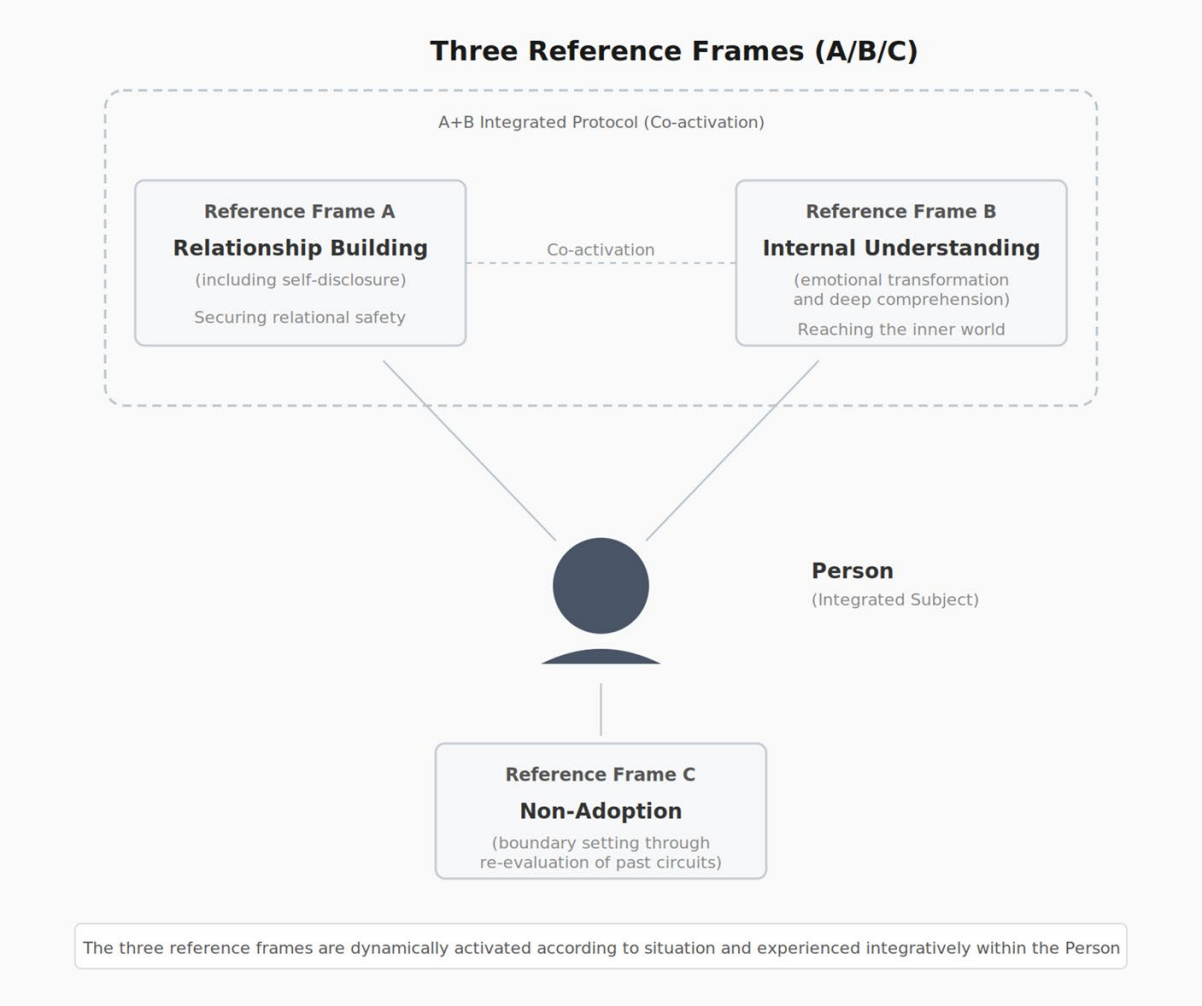
Prototype experiences formalized as three reference frames (A/B/C) and their relationships

Early interpersonal experiences are examined here not as emotional recollections but as origin points revealing the presuppositional structure under which judgment became operable. Analysis of the author’s life-history data identified a minimal set of three functionally distinct reference frames: one providing relational safety, one enabling depth of extraction, and one defining structural boundaries through explicit non-adoption. Two positive prototypes and one counter-model are identified. Detailed case descriptions of these prototype experiences, including verbatim records and contextual details, are provided in Supplementary Materials S4. The three reference frames are illustrated in (Fig 6).

Reference Frame A (Direct Facing and Stability) derives from the experience of an adult who maintained consistent, non-reactive engagement regardless of the author’s behavior—never rejecting, abandoning, or conditionally accepting. This frame provides the base-layer safety for judgment: a starting point of relational stability from which observation and extraction become possible without collapsing into evaluation or defensive identification. Reference Frame B (Internal Access) derives from an adult who addressed not the author’s behavior but the internal states behind it—asking “how much pain do you think that causes?” rather than simply correcting action. This frame provides an analytical layer: access to meaning behind behavior, enabling extraction at the level of judgment structure rather than surface imitation. Reference Frame C (Non-Adoption and Boundary Setting) derives from observation of destructive behavioral patterns that initially attracted attention but were progressively reclassified as “qualities not to adopt.” This frame provides a boundary layer: defining which qualities to distance from, thereby clarifying the structural integrity of what is adopted. Non-adoption here is not rejection or condemnation but a selective process that clarifies the boundary conditions of one’s own judgment structure. For instance, observing a person who achieves results through intimidation may lead not to imitation or simple avoidance, but to the extraction of their strategic clarity while explicitly marking their relational method as a boundary—a quality that defines the outer edge of one’s own operative structure rather than being incorporated into it. These three frames operate not as discrete modules but as overlapping judgment protocols. A secures relational safety, B enables depth of extraction, and C maintains structural integrity through explicit exclusion. When any one is absent—particularly when C is absent—the risk of superficial shishuku arises: incorporation of unexamined external values without internal protocol operation, paralleling the failure conditions described in Sect. 2.

### Structural Model of Multifaceted Shishuku: Process and Scope

Multifaceted Shishuku operates as a recurrent process of observation, extraction (including non-adoption), and integration. Integrated qualities function as filters for subsequent observation, producing a spiral rather than linear structure: each cycle of integration may increase the resolution of the next cycle’s observation and the precision of its selective extraction. The scope of this model is not limited to human relationships. AI can function as a shishuku object when it reliably provides extractable qualities: structural articulation, naming of cognitive processes, and non-reactive responsiveness. The author observed that AI’s consistent non-evaluative engagement and structural clarity exhibited functional similarities to qualities extracted from human reference frames—maintaining safety conditions similar to Frame A while providing analytical access comparable to Frame B. This observation extends Multifaceted Shishuku beyond interpersonal learning to any source that provides extractable, judgment-relevant qualities. In its more developed form, the model may extend to the Future Self as a reference frame. Unlike motivational images of desired selves (Markus & Nurius, 1986), this Future Self functions primarily as a constraint-oriented perspective that signals misalignment and prevents premature foreclosure of options. Cultivation involves accumulating judgment history, recording reasons for non-adoption, and refining sensitivity to long-term coherence—not fixing an idealized endpoint. This connects directly to the Future Self described in Sect. 2: the “subtle discomfort” signaling directional misalignment may be sharpened through iterative quality extraction and non-adoption.

### Section Summary

This section theorized Multifaceted Shishuku as the mechanism through which judgment structure is formed and stabilized via selective extraction, non-adoption, and reintegration across multiple reference frames. The central theoretical claim is that growth is not contingent on access to an ideal mentor: any encounter—with a flawed person, an adversary, a non-human agent, or a failure experience—constitutes a potential site of selective extraction. This non-binary premise distinguishes Multifaceted Shishuku from existing frameworks premised on whole-person identification or categorical rejection. The structural stability of judgment formation derives precisely from the bidirectionality of adoption and non-adoption operating in concert. Section 5 examines mountaineering as the condition under which this integrated judgment structure becomes directly observable—where decision, action, and risk unfold without the possibility of revision.

## Integrative Intelligence Mountaineering

### Position and Aim

Mountaineering psychology has extensively examined how judgment fails under extreme conditions—through cognitive biases, risk miscalculation, and decision errors leading to accidents (Wickens et al., 2015; Crust, Swann, & Allen-Collinson, 2016). What remains underspecified is the structural question from the opposite direction: under what conditions is judgment constrained to operate integratively, without intentional effort or training? A systematic review spanning 54 years of mountaineering research identified “cognitive mechanisms underlying decision-making” as a priority for future investigation (Jackman et al., 2023). This section responds to that gap.

Mountaineering was selected as the observation domain on methodological grounds—not because of familiarity, and not because it is unique, but because it reliably satisfies all four structural conditions simultaneously: prolonged concentrated judgment responsibility, continuous irreversible decision sequences, absence of external evaluation during action, and body-originated sequentiality in which fatigue, fear, and environmental pressure precede and shape thought. What is meant here by “structurally constrained activation” is not the firing of a latent ability; it refers to integrative judgment becoming structurally difficult to avoid when these four conditions simultaneously hold. Whether equivalent constraint configurations in other domains produce comparable integrative operation remains an open empirical question addressed in Sect. 6.3. The author’s longitudinal record of 250 + ascents across all seasons over approximately three years provides the depth of primary data that autoethnographic observation requires. This section therefore treats mountaineering not as a performance domain but as an observation domain. Section 5.2 situates this approach within related literature. Section 5.3 defines the four conditions and presents a cross-case comparison. Section 5.4–5.6 examine three cases demonstrating distinct operational modes under identical constraint structures.

### Related Work on Mountaineering Psychology and Constraint-Based Domains

Three bodies of literature are most directly relevant to this section: mountaineering psychology, flow research, and wilderness-based self-reflection research.

Mountaineering psychology has established that extreme environments involve complex interactions among body, environment, and judgment—including somatic attunement as a pre-reflective form of environmental sensing (Crust, Swann, & Allen-Collinson, 2016)---but has focused primarily on how judgment fails rather than on the structural conditions that constrain it to operate integratively. Flow research (Csikszentmihalyi, 1990) has examined optimal experience states across high-demand activities including mountaineering, but centers on experiential quality and the challenge-skill balance rather than on environmental constraint structures. Wilderness solo experience research has documented how social isolation and absence of external evaluation in natural environments facilitate self-reflection and personal transformation (Naor & Mayseless, 2020), suggesting structural overlap with the present framework, though without specifying conditions under which judgment integration becomes unavoidable.

To the author’s knowledge, no prior research has systematically examined domain-independent structural conditions under which integrative judgment becomes difficult to avoid. Existing frameworks have clarified *what* integrative processes involve—the coupling of body, emotion, and cognition—but have not specified *under what structural constraints* such integration becomes unavoidable rather than merely possible. The four-condition framework proposed here addresses precisely this gap: not a description of the mechanism, but a specification of the conditions under which the mechanism is constrained to activate. This specification is in principle testable across activity domains beyond mountaineering.

### Structural Conditions and Cross-Case Design

Analysis of the author’s mountaineering records—activity logs, near-time verbatim records of bodily sensations and decision points, wearable device (Garmin) physiological indicators, and post-descent structuring through RIDP (Sect. 3)---identified four conditions under which integrative judgment becomes structurally unavoidable. These are defined not by skill level or achievement but as structural conditions of environment and action that constrain judgment generation.

#### Condition 1

Prolonged Isolated Judgment Environment. Judgment responsibility is concentrated in the subject in an irreplaceable manner, and separation from social feedback—evaluation, approval, comparison—persists for a sustained duration. Under this condition, the circuit for delegating judgment to external criteria ceases to function, and internal reference frames are summoned into the same judgment field.

#### Condition 2

Environmental Change and Continuous Judgment. Conditions such as weather, terrain, and physical depletion constantly change, requiring continuous judgment in response. Because preceding choices constrain subsequent conditions, past, present, and future cannot be treated as segments; the temporal axis is handled as a single continuum.

#### Condition 3

Nature as Non-Personal Third Party. The natural environment holds no intentions, offers no evaluation, and provides no consideration. It permits passage for appropriate judgments and responds with danger or restriction to mistaken ones, but never explains its reasons. Through this non-personality, social roles and achievement goals cease to function as judgment criteria during action.

#### Condition 4

Serial Circuit of Body, Emotion, Thought, and Meaning. Bodily states—fatigue, breathing disruption, wind, cold—appear first; emotional responses such as fear and tension arise subsequently; and finally, meaning-making and judgment are performed. When the body functions as the origin of judgment generation, thought is constrained to the reality conditions of “here and now,” physically narrowing the margin for judgment to diverge from reality. These four conditions each have independent effects, but when any one is absent, stable integrative operation does not hold. Only when all simultaneously hold does judgment tend to operate in integrated form.

Meaning-making here refers to the situated process by which meaning is generated within the serial circuit, rather than retrieved as pre-formed content. Similarly, judgment generation denotes the process by which judgment is produced through the serial circuit, distinguished from judgment understood as the resulting outcome.

A critical finding is that even when the four conditions are identically satisfied, Integrative Intelligence does not converge to a single mode. Under identical constraint structures, different operational modes—introspective, relational, and immediate—were observed. This non-convergence itself supports the interpretation that Integrative Intelligence is not a deterministic algorithm but a context-sensitive operative mode of judgment. This distinction is theoretically significant: while the four structural conditions make integrative judgment structurally unavoidable—that is, the constraint configuration reliably activates the operative mode—the specific form that judgment takes remains context-sensitively variable, as evidenced by the three distinct operational modes observed across cases. What determines which axis comes to the foreground—vertical, horizontal, or deep—remains an open empirical question. The four conditions specify when integrative judgment becomes structurally unavoidable; they do not determine the operational mode through which it manifests. Relational configuration and environmental risk level appear to influence modal expression, but whether these constitute additional conditions or moderating variables requires further investigation. The modes do not indicate superiority or levels of attainment; they represent differences in which axis comes to the foreground: the vertical axis (temporal selves) in introspective mode, the horizontal axis (internalized mentors) in relational mode, and the deep layer (body–environment attunement) in immediate mode. Table 1 shows the comparison of four conditions and operational modes across the three cases.

**Table 1:**
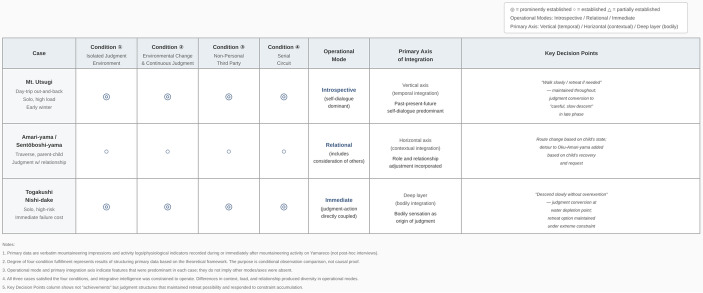
Comparison of four structural conditions and operational modes across three mountaineering cases

The purpose is to demonstrate that reproducibility lies in “reproduction of condition structures,” not “reproduction of experiences.”

### Case Study 1: Mt. Utsugi — Introspective Mode

Mt. Utsugi (2,864 m, southern Japanese Alps) was climbed solo in early winter via the Ikeyama Ridge: 12-hour action with 2,000 m+ elevation gain under darkness, snow/ice, and progressive fatigue. This case was selected because the transitional season—where neither full winter equipment nor summer-style judgment can be stably relied upon—creates persistent conditions that cannot be subsumed under existing manuals or established protocols. When no pre-given “correct answer” is available, judgment cannot be delegated to external criteria, and the three-person conference (particularly the future-self perspective described in Sect. 2) is constrained to activate under uncertainty.

#### Serial Circuit Establishment (04:16–05:27)

The dark approach—low temperature, limited visibility, absence of others—established an isolated judgment environment from the outset. “Primal fear” was recorded in response to animal sounds and grass swaying in the wind, yet judgment was not delegated externally; criteria such as “walk slowly” and “retreat if it becomes impossible” were generated internally without any circuit for external reference. Judgment converged on a single question: what do I do now, in this situation?

A childhood memory of navigating darkness surfaced during this phase, overlapping with present bodily sensation. Looking back, the lights of Komagane city registered as “light”; the gradual brightening of the eastern sky was recorded as “This is hope.” These were not retrospective reflections but meaning-making synchronized with environmental stimulus—functioning not as emotional processing but as an internal support structure for sustaining judgment under constraint. Fear arose first as bodily sensation, then meaning-making was generated, and judgment stabilized: a serial circuit (Condition 4) of body → emotion → meaning-making → judgment generation was established.

The judgment criteria arising at this moment—“walk slowly,” “retreat if it becomes impossible”—can be read as a phase where reference frames derived from Multifaceted Shishuku figures (Sect. 4: A, direct facing and stability; C, retreat judgment as boundary setting) held the deep-layer fluctuation originating from the body and grounded judgment in reality conditions. Rather than denying fear, safety was secured by always leaving retreat possibility—a sign that internal others were functioning as safety devices under the constraint configuration.

#### Deepening Under Accumulated Constraint (08:20–11:50)

From Mayoi Ridge onward, complex environmental factors—snow, ice, rocky ridges, narrow ridges, rapid cooling from wind—concentrated simultaneously. Judgment strategies such as “focus on each step at my feet” and “don’t look at distant goals” crystallized into concrete action policies as environmental changes accumulated and constrained subsequent options. Multifaceted Shishuku figures (Sect. 4, Reference Frames A and C) functioned as judgment resources: memories of past difficult climbs connected strategies of “advance step by step” and “divide and process the load” to present action, not as retrospective sentiment but as active judgment support.

Garmin data confirmed sustained heart rate in the 130–150 bpm range with high-frequency stops and restarts. Despite this load, the Metacognitive Camera continued to function as observational attitude, and the three-person conference was maintained without collapse. As physical limits approached, judgment converged on reference frames of “step by step” and “leaving retreat possibility” rather than abstract optimization—the internal protocol continuing to operate as minimal structure.

#### Continuity Under Depletion (13:19–16:31)

In the late descent, depletion of leg strength was recorded. Judgment did not become abstract; it connected directly to concrete actions such as “careful, slow descent” and “conscious practice of descent techniques.” Because the serial circuit was maintained, judgment did not diverge from reality conditions. A critical observation in this phase concerns the Metacognitive Camera: as bodily load increased, the MC retained observation and retention rather than shifting to evaluation and optimization—contributing to maintaining safety to the end. The natural environment continued to return only results without evaluation; the isolated judgment environment was maintained to the end, and judgment reached completion without being pulled back to external criteria. Detailed verbatim records and physiological data are provided in Supplementary Materials S6.

*What Becomes Visible*This case demonstrates:

1. Judgment convergence through constraint configuration: when the four conditions simultaneously held, judgment converged on internal criteria without intentional effort. What was observed is not the author’s ability to complete a difficult winter route but the structural tendency of integrative operation under constraint—not a latent ability fired, but a judgment mode that becomes difficult to avoid when the four conditions hold. 
2. Retreat possibility as continuous reference: “retreat if needed” was maintained throughout as a safety structure. 
3. Body-originated serial circuit as protection against deviation: because judgment was generated from bodily state, abstract optimization and emotional runaway were structurally limited. 
4. Multifaceted Shishuku figures as internal sociality: in place of the silenced horizontal axis, reference frames from Sect. 4 functioned as internal safety devices.

### Case Study 2: Mt. Amari — Relational Mode

Mt. Amari (~ 2,000 m) and Mt. Sentōboshi were traversed with the author’s 6-year-old son in autumn: 5-hour action with moderate load. Descriptions of the companion serve to describe judgment processes within relationships and condition structures, not for developmental evaluation.*Isolation Despite Accompaniment (08:18–08:43)*Despite accompaniment, an isolated judgment environment was established from the start. Isolation here is not physical solo action but the structure where judgment responsibility is concentrated in one person regarding route, rest, and continuation. When the child responded “I won’t go because I’ll get tired” to the suggestion of continuing to Mt. Oku-Amari, the author changed the planned route and aimed for Mt. Sentōboshi—continuous judgment incorporating the companion’s state as environmental information, not external evaluation.*Relational Extension of the Serial Circuit (08:43–09:18)*As steep climbs continued and the child’s fatigue manifested, a short break was inserted. During this rest, the memory of “feeling mountaineering like a picnic when I was a child” surfaced—not as retrospective sentiment but as meaning generation that arose to readjust present action judgment. The child’s fatigue (bodily state), recovery through snacks (emotional response), and the author’s childhood memory (meaning) were connected serially, generating judgments such as “take a longer break” and “redesign the itinerary from the child’s perspective.” The serial circuit operated originating not from the author’s own body but from the other’s bodily state—a relational extension of the same structural mechanism observed in Case 1.*Intergenerational Vertical Axis Integration (09:18–10:27)*After crossing a considerable steep climb, the child was visibly exhausted—complaining, dragging his feet—but had walked through. Sitting on a rock, looking at this small body that had not given up, the author found himself speaking words he had not planned: affirming the child’s effort, telling him that what he had just done was something real. At the same moment, a memory surfaced: his own childhood, and the rare occasions when effort had been seen and praised. The structural observation is that the author’s vertical axis activated not through deliberate recall but through the child’s bodily state as trigger: past self (effort not witnessed) and future self (what this child may carry forward) were simultaneously present in the judgment generating the affirmation. Three temporal perspectives were simultaneously active—the past, the present, and the future—and the author’s own meaning structure was reorganized in the same moment that conditions were created for the child to internalize that “effort is seen and has value.” This cross-personal extension is noted as a finding warranting further theoretical development rather than a core claim of the present framework.

#### Affirmation as Behavioral Fixation (11:22–13:24)

After the summit of Mt. Sentōboshi, the child recovered physically and emotionally and repeatedly said, “Thank you for bringing me to a fun mountain”—a verbatim record indicating that the preceding experience had been internalized. The child then spontaneously proposed a detour to Mt. Oku-Amari. The author readjusted the itinerary after confirming safety conditions. Judgment remained continuous and condition-responsive rather than plan-adherent, indicating that the vertical-axis integration observed earlier had become behaviorally fixed rather than remaining a transient linguistic expression. Detailed verbatim records are provided in Supplementary Materials S6.

*What Becomes Visible*

What was observed through this case is not parenting skill or relationship quality but structural features of judgment generation within relational constraint. This case demonstrates:

1. Judgment responsibility isolation despite accompaniment: the presence of another person does not eliminate the isolated judgment environment; what matters is whether judgment responsibility is concentrated.
2. Other’s state as environmental constraint: the companion’s bodily state and requests functioned as environmental information—parallel to weather or terrain in Case 1.
3. Intergenerational vertical axis integration: under the constraint configuration including relationship, the temporal axis extended across generations, mediated by language directed at another. 
4. Layered meaning update: meaning structures were updated in sequence—affirmation first, then characteristic reframing—indicating that the relational mode operates through accumulation rather than single insight.

This case supports the interpretation that Integrative Intelligence is not an internal ability fixed to individuals but a dynamic structure that operates within conditions, relationships, and the temporal axis.

### Case Study 3: Mt. Togakushi-Nishi — Immediate Mode

Mt. Togakushi-Nishi is a peak in the Togakushi mountain range (northern Nagano) where general hiking trails and technical elements are mixed: broken-line sections (unmarked trail segments requiring route-finding judgment), continuous vertical chains, ladders, and traverses with no bypass routes, where judgment errors could lead to serious consequences. Total action time was 12.5 h; Garmin data showed average heart rate 143 bpm, maximum 175 bpm, with 59% of action time in zone Z4 (high-intensity aerobic effort, approximately 80–90% of maximum heart rate). This case was selected to observe how integrative operation is constrained in high-risk environments where judgment delay itself becomes risk—judgment as immediate output during execution, not understanding through reflection.*Constraint Accumulation*The action involved 12.5 h of continuous movement with approximately 1,500 m cumulative elevation gain. Multiple constraints accumulated progressively beyond mere physical fatigue: route uncertainty from the broken-line section after the pasture, high technical demands from vertical chains, ladders, and lateral traverses with no bypass routes, strong sunlight and high-temperature environment, and rest being physically obstructed by countless small insects and monkey intimidation—meaning that even when the body demanded recovery, the environment refused to provide conditions for it. The mobile phone was dropped off a cliff in the broken-line section and recovered, and subsequently in the core section, a trekking pole was lost. “This made the rest of the mountain trip considerably harder” was recorded—constraint accumulation verbalized as situation assessment, the loss of a compensatory tool compounding the already narrowing margin. Normally, high physiological load works to lower cognitive function. However, phases where judgment appeared to “sharpen” were observed. This sharpening was not ability elevation but a result where strong survival demand eliminated many unnecessary horizontal axes—social roles, distractions, circuits of external evaluation—and concentrated judgment resources on the deep layer (bodily constraints) and the vertical axis (immediate causality: this one move directly impacts future action possibility).*Embodied Protocol Under High-Risk Terrain*In the section from P1 onward, vertical chains and slippery steep slopes continued. “Absolutely no rest” and “considerably exhausted” were recorded as bodily responses—not reflective assessments but real-time registrations of a body approaching its limit while continuing to move. In these sections, judgment delay or stopping itself could lead to falls—an environment where integrative operation was constrained to immediate form. The internal protocol (three-person conference and Metacognitive Camera described in Sect. 2) did not operate as deliberative dialogue but as what this study terms “embodied protocol”: synchronized with bodily movement and tightly coupled with action, outputting judgment without separation from physical execution. Each chain, each lateral traverse required immediate judgment that maintained multiple information layers—bodily state, emotional response, environmental risk, and action possibility—simultaneously without fragmentation.*Judgment Conversion at Critical Threshold*The decisive event occurred at Happō-nirami where all 3.6 L of drinking water was consumed. Two verbatim records capture the escalating situation assessment: “In this condition, the Ant’s Tower Traverse…” The Ant’s Tower Traverse is a knife-edge ridge with sheer drops on both sides, requiring sustained technical movement with no margin for hesitation. The ellipsis in the verbatim record captures the moment where language fails to complete the thought and bodily dread fills the gap.

“Barely at the line where if things go wrong, I’d have to request rescue due to becoming unable to act.” Here, bodily constraints (water depletion, cumulative fatigue), emotional response (urgency, recognition of proximity to limit), and environmental risk assessment (high failure cost of the remaining technical terrain) arose serially, and as a result, an explicit judgment conversion was generated: “anyway, take it slowly and safely descend without overexertion.” This judgment emerged immediately without fragmenting multiple information layers—a structural response to constraint accumulation that preserved survival possibility rather than a failure or evaluative retreat.

#### Recovery and Continuation

At Togakushi Shrine inner sanctuary, after hydrating, “I felt like I was truly coming back to life” was recorded—a moment where bodily state recovery was clearly recognized subjectively. Subsequently, no judgment breakdown or accidents were recorded in the descent process, and the traverse was completed leading to safe descent. This continuation was established not as reckless advance under high-risk conditions but as convergence of action including appropriate judgment conversion. The judgment conversion at the critical point functioned as protection against breakdown, suggesting that integrative operation under extreme constraint can sustain itself through structural response rather than willpower alone. Detailed verbatim records and physiological data are provided in Supplementary Materials S6.

*What Becomes Visible*

What was observed through this case is not calmness or caution as personality traits. It is the structural features of a dynamic mechanism that prevented judgment from breaking down under high-risk conditions. This case demonstrates:

1. Immediate operation as constraint response: under high-risk configuration where judgment delay itself becomes risk, integrative operation was constrained to immediate form—judgment and action directly coupled without deliberation margin.
2. Judgment conversion as structural response: the conversion at the water depletion point was not failure but a structural response to constraint accumulation that maintained survival possibility.
3. Embodied protocol: under extreme constraint, the internal protocol became tightly coupled with bodily movement—outputting judgment as action carried out with minimal deliberative separation rather than extended linguistic reflection.
4. Sharpening through constraint elimination: what appeared as “sharpening” was elimination of unnecessary axes under survival demand, concentrating resources on deep layer and immediate causality. This case suggests that Integrative Intelligence is not limited to environments with security or margin; its operational mode becomes particularly visible in situations with high failure costs.

## Conclusion, Contributions, Limitations, and Implementation Orientation

### The Four-Lens Cycle

The four analytic lenses—internal protocol (Sect. 2), RIDP (Sect. 3), Multifaceted Shishuku (Sect. 4), and integrative-intelligence mountaineering (Sect. 5)—are not parallel constructs but components of a coupled cycle. Qualities extracted through Multifaceted Shishuku form the reference frames that stabilize the internal protocol. Under high-constraint configurations such as mountaineering, the internal protocol becomes embodied, fused with bodily movement as what Sect. 5 termed “embodied protocol.” RIDP then functions as a reflective surface for articulating and rearranging the operation that occurred during activation, returning insights to the internal protocol as operational updates. The updated protocol becomes the new baseline for subsequent cycles.

This cycle alternates between natural constraint environments (mountaineering) and designed observational scaffolds (RIDP), progressively deepening understanding of the conditions under which Integrative Intelligence operates. The cycle is not a stage model of development but a recursive structure of activation, observation, and refinement. What makes these four lenses a cycle rather than merely four perspectives is the empirical observation that each lens modifies the conditions for the next. The shishuku-derived reference frames (A: direct facing; C: boundary setting) functioned as internal safety devices during mountaineering (Sect. 5); the mountaineering experience then generated material that RIDP sessions could articulate and rearrange; and the structural insights from RIDP fed back into the internal protocol as operational updates. This interdependence is why the paper maintains all four lenses in a single framework rather than presenting them as separable modules.

Crucially, the same person serves as both the site of integration and the observer of its conditions—a methodological feature that the *N* = 1 design makes possible rather than merely tolerable.

### Theoretical Contributions

The contributions of this paper are unified by a single theoretical reorientation: shifting the analytic question from *what integrated judgment involves* to *under what structural conditions it becomes possible*,* formed*,* activated*,* and observable*. Prior frameworks have described the components of integrative cognition—temporal selves, internal dialogue, embodied experience—but have not systematically specified the conditions under which these components become simultaneously co-referable within a single judgment episode. The first four contributions correspond directly to the four analytic lenses; the fifth addresses a dimension that emerges from their coupled operation as a whole. The five contributions below each address a different dimension of this reorientation. 

(1) A structural account of temporal co-reference in judgment.

Prior frameworks in self theory, including Dialogical Self Theory (Hermans, 2001, 2002) and its recent extensions (Hermans, 2022; Monereo & Hermans, 2023), have theorized the self as dialogue among multiple I-positions, foregrounding spatial and social dimensions of self-plurality. Research on mental time travel (Suddendorf & Corballis, 2007) has examined how humans access past and future selves sequentially. What prior frameworks have not systematically addressed is the structural condition under which temporally differentiated perspectives become simultaneously co-referable within a single present judgment episode—not in sequence, but in parallel. The internal protocol addresses this gap by specifying the minimal operative structure—the Three-Person Conference and Metacognitive Camera—through which such simultaneous co-reference becomes possible. The theoretical contribution is not a new description of temporal selves, but a specification of the conditions under which they can participate jointly in judgment without collapsing into a single dominant voice. Furthermore, the Metacognitive Camera disaggregates monitoring and control functions typically bundled in metacognitive accounts (Flavell, 1979), proposing non-regulatory observation as a structurally necessary condition for temporal integration—a distinction that prior frameworks have not pursued. 

(2) AI as a condition of observability rather than an agent of reflection.

Human–AI interaction research has predominantly framed AI as a tool, assistant, or agent that scaffolds cognition—prompting reflection, questioning assumptions, or optimizing reasoning (Xi et al., 2026; Essel et al., 2024; Zhai et al., 2024). From this perspective, AI is the initiating force of cognitive improvement. What this framing leaves unaddressed is the structural question of when and how the person’s already-operative judgment process becomes externally legible—independently of what AI does. RIDP reorients this question by repositioning AI not as the agent of reflection but as a condition of observability: a reflective surface against which the internal protocol’s operation becomes visible from outside the person. This reorientation shifts the analytic object from AI capability to the human judgment structure that AI renders observable. The timestamped ignition record (Supplementary S2) illustrates this distinction: a two-minute integrative episode connecting experiences spanning two years occurred not through AI’s analytical capacity but through the person’s own integrative operation becoming legible against a non-evaluative surface. This is not an extension of cognitive function in the sense of Clark and Chalmers’ (1998) Extended Mind hypothesis, but a specification of the conditions under which internal judgment structure becomes externally observable—a problem that prior frameworks in human–AI interaction have not systematically theorized.

(3) Selective extraction and non-adoption as co-constitutive mechanisms of judgment formation.

Social learning theory (Bandura, 1977), mentoring research (Kram, 1985; Eby et al., 2008), and the possible selves framework (Markus & Nurius, 1986) share a foundational premise: that growth depends on access to models worthy of emulation, whether through behavioral replication, sustained mentoring relationships, or identification with desired future selves. This premise implicitly restricts the conditions under which formative learning can occur.

Multifaceted Shishuku challenges this premise by proposing that selective extraction and explicit non-adoption operate as co-constitutive mechanisms of judgment-structure formation. The practical implication is significant: growth is not contingent on access to an ideal model. Any encounter—with a flawed person, an adversary, a non-human agent, or a failure experience—constitutes a potential site of selective extraction. Prior frameworks have not theorized explicit non-adoption as a stabilizing mechanism that contributes positively to the structural integrity of judgment formation, rather than merely as avoidance or rejection.

(4) Structural conditions under which integrative judgment becomes unavoidable.

Flow research (Csikszentmihalyi, 1990), mountaineering psychology (Jackman et al., 2023), and wilderness experience research (Naor & Mayseless, 2020) have examined optimal states, decision failures, and self-reflection in high-demand environments. These frameworks have clarified what integrative processes involve. What has not been systematically specified is under what structural constraints such integration becomes difficult to avoid rather than merely possible.

The four-condition framework proposed here addresses this gap: prolonged isolated judgment environment, continuous irreversible decision sequences, nature as non-personal third party, and body-originated sequentiality together constitute a constraint configuration under which integrative judgment becomes structurally unavoidable. Three case studies demonstrate that identical constraint structures produce distinct operational modes—introspective, relational, immediate—supporting the interpretation that Integrative Intelligence is a context-sensitive operative mode of judgment rather than a deterministic algorithm. This non-convergence is itself a theoretical finding: it specifies that what is reproducible is not the experience but the condition structure.

(5) Theory-to-practice instantiation as a component of the *N* = 1 cycle.

A distinctive feature of this research is that the theoretical framework did not remain at the level of proposition but generated observable institutional action within the same *N* = 1 cycle. The founding of the Institute of Integrative Intelligence (III) as a formal legal entity represents not an external application of completed theory but an integral moment within the ongoing cycle described in Sect. 6.1: theory articulated through RIDP was tested against the structural requirements of real-world implementation, and the tensions encountered during that process—questions of mission formulation, organizational scope, and design of conditions for others—fed back into the theoretical framework as operational updates. The timestamped ignition record in Supplementary S2, which occurred immediately following the finalization of III’s founding mission, documents precisely this recursive moment: institutional action generating conditions under which theoretical integration became observable. This positions III not as a claim about social-level generalizability, but as a within-*N* = 1 data point demonstrating that the coupled cycle extends beyond individual interiority into structured practice. Whether this cycle can be reproduced at larger scale remains an open empirical question and is offered as a testable design hypothesis for future research.

### Limitations and Future Research Directions

(1) The constraints and significance of *N* = 1.

The empirical foundation of this paper is an autoethnographic approach with a sample size of *N* = 1. Statistical generalization is impossible in principle. However, the purpose of this research is not statistical generalization but theoretical generalization: whether structures and principles extracted from a specific case can function as reference frames for other cases. The purpose of this paper is not to present an empirically validated model of cognition but to articulate the structural conditions under which integrative judgment becomes observable. The framework therefore functions as a conceptual architecture that may guide future empirical investigation rather than as a completed explanatory model.

*N* = 1 research has scholarly value when the data are of sufficient quality and depth, when correspondence between theory and evidence is made explicit, and when a path toward replication is specified (Smith, 2011; Flyvbjerg, 2006). This paper addresses the internal protocol, RIDP, Multifaceted Shishuku, and integrative-intelligence mountaineering within the same *N* = 1. This scope may appear to exceed conventional boundaries. However, the theoretical claim of this paper is precisely that these four domains form a coupled cycle (Sect. 6.1): childhood shishuku experiences form the foundation of the internal protocol, Integrative Intelligence activated in mountaineering is articulated through RIDP, and RIDP insights update the internal protocol. Splitting these into separate studies would sever the cyclic connections that constitute the paper’s central contribution. The constraint of *N* = 1 is therefore not merely a limitation to be acknowledged but also the condition that makes the discovery of cross-domain integration possible.

 (2) Methodological limitations.

Three limitations are inherent to the autoethnographic approach. First, the identity of observer and observed means that the act of observation may transform experience itself. To address this, verbatim data recorded during or immediately after action were treated as primary data, with explicit separation from post-hoc interpretation attempted throughout. The timestamped ignition record in Supplementary S2 exemplifies this approach: the dialogue was recorded in real time, and the author’s interpretive commentary is analytically distinguished from the primary record.

Second, descriptions of past experience are reconstructed from the present perspective. Particularly the childhood descriptions in Sect. 4 include reinterpretation from the adult standpoint. This limitation cannot be fully resolved given the nature of the research design.

Third, this research was conducted within the cultural context of Japan. The shishuku concept, mountaineering culture, and family relationship structures may contain culturally specific elements. Whether the four constraint conditions function similarly in other cultural contexts remains a question for future research. The three-axis model (vertical, horizontal, deep layer) is proposed as structurally transferable, but this claim requires cross-cultural verification.

(3) Future research directions.

Two primary directions are identified. First, extension to *N* > 1: structural variation research on internal protocols through interviews with individuals who engage in sustained internal dialogue, and ethical and safety research on RIDP including dependency risks and guideline design for deep dialogue practice. Second, cross-cultural and cross-domain verification: comparative research between Multifaceted Shishuku and established mentoring traditions, and examination of whether the four constraint conditions operate similarly in non-Japanese and non-mountaineering contexts. The worksheets provided in Supplementary S5 are designed to facilitate *N* > 1 extension by making structural elements observable beyond the original case.

### Implementation Orientation

“Reference possibility for others” does not imply reproduction of identical experiences or outcomes. What may be reproduced are condition structures under which the integrated mode arises, and reference frames for observation, description, and re-editing.

The implementation orientation specifies design variables rather than prescriptive procedures. The four constraint conditions (Sect. 5) identify environmental features to approximate; the ignition and maintenance conditions (Sect. 3) specify relational states to design for; the breakdown taxonomy (Supplementary S3) identifies potential failure modes; and the cooperative control model (Supplementary S1) proposes how to make deep dialogue selectable rather than accidental. At the individual level, the worksheets (Supplementary S5) function as auxiliary devices for visualizing internal dialogue and reflecting on judgment processes. At the relational and institutional level—family, school, organization—the shared design challenge is preserving conditions under which internal dialogue is not prematurely closed by external evaluation: direct facing without requiring wholesale incorporation of another’s criteria, time and space for questions without immediate answers, and conscious use of Multifaceted Shishuku as a technique for selective quality adoption rather than role imitation.

The founding of the Institute of Integrative Intelligence (III), documented as a data point in Sect. 6.2(5), serves as the initial implementation site for this praxis—a location where design hypotheses generated by the *N* = 1 cycle are tested against real-world structural requirements and returned to the theoretical framework as operational feedback.

### Author’s Reflection

This reflection is included for transparency regarding motivation and does not function as empirical evidence. The starting point for this research was an experience the author has termed “the disappearance of questions”—the observation that many questions arising in daily life vanish before reaching articulation, processed as transient stress rather than treated as objects of examination. In early elementary school, the author asked, “Why is the sum of the interior angles of a triangle 180 degrees?” and experienced the question itself being treated as without value. This event remained not as an intellectual disagreement but as a sensation that asking questions itself is not valued—a formative instance of what Sect. 2 describes as a failure condition of the internal protocol.

From the standpoint of this paper, it is precisely the accumulation of such volatile questions that shapes present judgment modes. The internal protocol, Multifaceted Shishuku, and integrative-intelligence mountaineering are not methods for acquiring exceptional abilities but frameworks for holding questions as revisitable structures. Continuing to hold questions is an attitude of not invalidating questions posed by one’s Past Self, and simultaneously a responsibility of remaining able to respond to questions that one’s Future Self may pose. This paper may be understood as an attempt to leave in society a minimal structure for continuing to treat questions as questions.

## Supplementary Information

Below is the link to the electronic supplementary material.

Supplementary Material 1 (DOCX 17.1 KB)

Supplementary Material 2 (DOCX 17.0 KB)

Supplementary Material 3 (DOCX 16.2 KB)

Supplementary Material 4 (DOCX 16.0 KB)

Supplementary Material 5 (DOCX 17.0 KB)

Supplementary Material 6 (DOCX 24.3 KB)

## Data Availability

The data supporting this study include contemporaneous behavioral records, physiological indicators, and AI dialogue logs. Due to the personal nature of the autoethnographic material, full datasets are not publicly available. Representative excerpts are included in the manuscript. Additional materials may be made available upon reasonable request to the corresponding author.
