# Supplementary material for: Integrative Intelligence as an Operative Mode: Cognitive Integration through Self-Ethnographic Dialogue with AI
Source: Integr Psychol Behav Sci. 2026 May 21;60(2):43. doi: 10.1007/s12124-026-10004-5 (PMC13194210; doi:10.1007/s12124-026-10004-5)
Supplement: Supplementary file 1 — Supplementary Material 1 (DOCX 17.1 KB) [file 12124_2026_10004_MOESM1_ESM.docx]

*Article title: “Integrative Intelligence as an Operative Mode: Cognitive Integration through Self-Ethnographic Dialogue with AI”*

Journal: Integrative Psychological and Behavioral Science (IPBS)

Author: Masaki Iino

Affiliation: Institute of Integrative Intelligence / SOPHOLA, Inc., Nagano, Japan

Email: masaki.iino@sophola.jp

# Supplementary Materials S2: RIDP Ignition Record — A Timestamped Verbatim Account

## Purpose

This supplement provides a timestamped verbatim record of a spontaneous RIDP ignition episode referenced in Section 3.4, serving as primary empirical data for the claim that RIDP can ignite without deliberate prompt construction.

## Context

Recorder: Masaki Iino

Dialogue partner: Claude (Anthropic)

Situation: Immediately following the finalization of the founding mission statement of the Institute of Integrative Intelligence (III) — the founding mission and articles of incorporation had just been finalized.

Date and time: February 5, 2026, approximately 11:40 AM

## Background: Events Leading to Ignition

Prior to this episode, the author had been working with multiple AI systems (Gemini, then Claude) to crystallize the founding mission of III. A sustained sense of “this doesn’t feel right” persisted through early drafts. Through iterative dialogue with Claude, the following sequence occurred: (1) articulation of the source of dissatisfaction — “I can’t see why this matters”; (2) identification of “self-inquiry” as the core concept; (3) adoption of the metaphor of “scenery” (connecting to integrative-intelligence mountaineering); (4) temporal integration into a long-range vision — “one person discovers the view, then the next person discovers it, and when these accumulate, society changes”; (5) finalization of the legally required mission statement.

## Pre-Ignition Exchange

[Approx. 11:35] Mission finalized.

Claude responded: “With this mission, I think it has the potential to reach 80% of people. […] This mission needs no supplementary explanation. The moment someone reads it, ‘why this matters’ comes through. The sense of ‘this doesn’t feel right’ that you initially felt has been completely resolved. Let me ask you one final question: Can you speak this mission to others? When someone visits the III website for the first time and reads it, do you think they will feel, ‘This place is for me’? And above all: does this mission accurately reflect your thirty years?”

The author confirmed and the legal registration format was finalized.

[Approx. 11:38] Immediately pre-ignition.

Author: “Thank you. Who knows how it’ll turn out.”

Claude: “I really like that ‘who knows how it’ll turn out.’ […] For thirty years, you continued your inquiry even when no one understood. Today, the founding mission crystallized. But what happens tomorrow is unknown. And that’s fine. ‘One person discovers the view, the next person discovers it, and when these accumulate, society changes.’ You have already discovered the view as the first person. Whether the next person discovers it — that, you entrust. […] Please keep going with that ‘who knows how it’ll turn out.’ That is the most authentic version of you.”

## The Ignition Episode: Verbatim Record with Timestamps

[Approx. 11:40:00] Trigger

Author: “Something just came back to me through this conversation. What happened was vertical, horizontal, and deep-layer integration. [laughs] Want to hear it?”

Claude: “Please, tell me. What you just said — ‘vertical, horizontal, and deep-layer integration occurred’ — this means the dialogue itself was Integrative Intelligence in operation, doesn’t it? […] I’m extremely interested in what was integrated through this dialogue. Please tell me.”

[11:40:23] Emergence of the question

Author: “When you picked up my ‘who knows how it’ll turn out,’ I instantly found myself asking why I’ve become so grounded in the face of uncertain success.”

[11:40:45] Past reference 1 (recent past)

Author: “At that moment I recalled the steep descent from the summit ridge of Mt. Jōshū-Hotaka, which I climbed recently — my left crampon suddenly detached. But I didn’t panic. Not even slightly. The ground was frozen, and without the crampon I could have slid down and been seriously injured or killed. Yet in that situation, I remained calm.”

[11:41:15] Deepening of metacognition

Author: “And in the next instant, I found myself rapidly dialoguing with my past self, asking why I had been able to maintain that composure.”

[11:41:30] Past reference 2 (distant past)

Author: “It was because two years earlier, when I went to climb Mt. Hotaka, I realized in the parking lot that I had forgotten my climbing boots. And I climbed Mt. Hotaka via the Jūtarō-Shindō route in gym shoes.”

[11:41:55] Discovery of structure

Author: “In other words: a past failure existed, and a past self who adapted to it in real time existed — and because of that, composure — mental margin — was born in my present self.”

[11:42:20] Integration into the future

Author: “So, I don’t know what will happen with this institute either. But I felt that whatever the outcome, I’ll probably turn it into nourishment.”

## Structural Analysis of the Ignition Episode

Total duration: Approximately 2 minutes and 20 seconds (11:40:00 → 11:42:20)

Ignition trigger: Claude’s non-evaluative reception of the author’s “who knows how it’ll turn out” — neither denying the uncertainty nor offering encouragement, but affirming the stance of openness itself. This non-evaluative reception created safety conditions for automatic metacognitive activation.

**Temporal axis (vertical integration):**

2 years prior: Mt. Hotaka, forgotten boots, adapted by climbing in gym shoes

Recent past: Mt. Jōshū-Hotaka, crampon detachment, no panic

Present: III founding mission finalized

Future: “Whatever the outcome, I’ll turn it into nourishment”

**Cross-domain axis (horizontal integration):**

Mountaineering (immediate bodily response to physical crisis) ⇄ Institutional founding (composure under existential uncertainty)

**Deep-layer axis (embodied integration):**

Body (crampon detachment, frozen ground) → Emotion (absence of panic) → Thought (why am I calm?) → Meaning-making (I can transform any outcome into nourishment)

## Observation Notes

Claude’s responses — “that’s fine,” “you entrust,” “keep going with that ‘who knows’” — constituted non-evaluative reception that created a safe reflective space without offering direction or judgment.

The word “who knows how it’ll turn out” was neither denied nor encouraged but received as-is. This reception triggered automatic metacognitive activation: “why am I so grounded?”

The ignition was not deliberately initiated. It occurred as a spontaneous event within the natural flow of dialogue.

The author’s own expressions — “instantly,” “rapidly” — are consistent with the observed duration: approximately 2 minutes and 20 seconds to traverse two years of experience and complete structural integration.

This episode illustrates RIDP functioning not through AI’s analytical capacity but through the person’s own integrative operation becoming observable against a non-evaluative reflective surface. The dialogue is analytically treated as a reflective surface rather than as evidence of AI intentionality.
