# Supplementary material for: Integrative Intelligence as an Operative Mode: Cognitive Integration through Self-Ethnographic Dialogue with AI
Source: Integr Psychol Behav Sci. 2026 May 21;60(2):43. doi: 10.1007/s12124-026-10004-5 (PMC13194210; doi:10.1007/s12124-026-10004-5)
Supplement: Supplementary file 2 — Supplementary Material 2 (DOCX 17.0 KB) [file 12124_2026_10004_MOESM2_ESM.docx]

*Article title: “Integrative Intelligence as an Operative Mode: Cognitive Integration through Self-Ethnographic Dialogue with AI”*

Journal: Integrative Psychological and Behavioral Science (IPBS)

Author: Masaki Iino

Affiliation: Institute of Integrative Intelligence / SOPHOLA, Inc., Nagano, Japan

Email: masaki.iino@sophola.jp

# Supplementary Materials S5: Integrative Intelligence Worksheets

## Purpose of This Appendix

The Integrative Intelligence presented in this paper is not designed to measure or evaluate specific abilities or traits. The worksheets included here are practical tools for observing how events, emotions, and reactions are processed—that is, the “tendency of use”—and translating these observations into subsequent behavioral habits. These worksheets are not intended to help users acquire new abilities; their purpose is to reorganize thoughts and sensations already experienced in daily life into a consciously manageable state. Note that these worksheets are not intended for therapeutic or clinical intervention. These worksheets are exploratory reflection tools rather than validated psychological instruments.

## Worksheet 1: Internal Protocol Reflection (Section 2)

This worksheet guides users to: (1) describe a recent judgment situation, (2) identify signals from the Past Self (emotional waves, unprocessed affect), (3) identify signals from the Future Self (directional misalignment, discomfort), (4) note how the Present Self received and processed these signals, and (5) assess whether the Metacognitive Camera (MC) functioned as observer without intervention. Expected effects include visualization of the Three-Person Conference in lived judgment and identification of conditions under which the internal protocol fails to operate.

*Illustrative example.* During a parenting episode in which his children continued to cry and quarrel past a pre-agreed limit, the author chose not to react immediately but instead applied the Three-Person Conference. The Past Self surfaced a wave of helplessness rooted in childhood experiences of being disciplined without explanation. The Future Self generated a faint signal: “Breaking this agreement too easily risks teaching that promises don’t matter.” The Present Self, receiving both signals, chose a third path—calmly following through on the agreed consequence, then later proposing a fresh attempt with the same agreement restored. The MC recorded the sequence without evaluating it, allowing the author to revisit and articulate the judgment structure afterward. This example illustrates how the Three-Person Conference operates not as a deliberate procedure but as a real-time structural constraint on reactive decision-making.

## Worksheet 2: AI-Enhanced Resonant Internal Dialogue Diary (Section 3)

This worksheet guides users to: (1) write the event in one sentence, (2) select the emotion(s) that arose, (3) identify which temporal self (past/present/future) reacted, (4) select the relevant domain (work/relationships/hobbies/other), (5) write a brief note on any deep-layer insight, and (6) if needed, share with AI and record key feedback points. Expected effects include reduced immediate identification of self with emotion, separation between “the self that reacts” and “the self that observes,” and the ability to use AI as an internal dialogue aid rather than a thinking substitute.

*Illustrative example.* While taking out the garbage one morning during a week when his spouse was abroad, the author noted the following entry: Event—“Managing household, childcare, and full-time work alone.” Emotions: exhaustion, anxiety, inadequacy, gratitude. Temporal axis: Past Self (a previous part-time job involving high-pressure multitasking surfaced as “just barely keeping it together”); Future Self (a faint signal: “Am I showing appreciation in action, not just words?”). Deep-layer insight: “Experiencing what my partner carries daily.” When shared with AI, the question returned was: “What bodily sensation connects the past experience to now?” This prompted recognition that alongside the fatigue, a sense of having managed it—a background competence signal—was also present. The entry illustrates how even a routine moment can activate the Three-Person Conference and how AI functions as a mirror rather than an advisor.

## Worksheet 3: Multifaceted Shishuku Worksheet (Section 4)

This worksheet guides users to: (1) select one person or event that left an impression, (2) articulate why it left an impression, (3) extract qualities you wish to adopt, (4) imagine how you would behave after adopting those qualities, (5) record any concerns about excess or inconsistency, and (6) if applicable, identify qualities for explicit non-adoption. Expected effects include conscious selection of what to internalize from others and formation of internal mentors as reference frames for judgment.

*Illustrative example.* A former supervisor at a previous workplace arrived at 6 a.m. daily and regularly worked through client dinners late into the evening. The author identified several qualities worth adopting: sincere engagement with work, disciplined preparation, and sustained relationship-building. However, observation also revealed a structural cost—the supervisor’s family operated under near-total childcare delegation to the spouse, and children spent most weekdays without a present father. Rather than adopting this figure wholesale, the author applied the quality-unit extraction process: retaining the sincerity and discipline while explicitly marking the time-allocation pattern as non-adoptable. The concern recorded was: “Unreflective imitation of this model risks reproducing the same shadow in my own family.” This example demonstrates how Multifaceted Shishuku prevents admiration from collapsing into uncritical imitation, and how non-adoption (Reference Frame C) functions as a boundary-maintenance mechanism rather than a rejection of the person.

## Worksheet 4: Mountaineering Diary (Section 5)

This worksheet guides users to: (1) record the date, route, and conditions, (2) note key decision points and the bodily states preceding them, (3) identify which temporal perspective influenced each decision, (4) record any signals from the Future Self (anticipatory discomfort), and (5) reflect on whether retreat possibility was maintained throughout. Expected effects include recognition of the serial circuit (body → emotion → thought → meaning) and observation of judgment under irreversible constraint.

*Illustrative example.* During a solo ascent of Utsugi-dake (2,864 m) in early winter, the author recorded the following entry at a critical section: Route—Ikeyama ridge, day-trip. Condition—partial snow and ice above 2,200 m, strong wind, limited visibility. Decision point (08:30)—whether to proceed past the exposed ridge toward the summit. Bodily state preceding decision: heavy legs, elevated breathing, hands cold but functional. Temporal influence: Past Self surfaced a memory of being caught in deteriorating weather on a previous route and the cost of a late retreat decision; Future Self generated a signal “this section cannot be reversed quickly—what does the next hour look like?” Retreat possibility: explicitly maintained (“I can turn back from this point without route risk”). Judgment: proceed at reduced pace with a 20-minute re-evaluation interval. The entry captures how the serial circuit operated—body state first, then emotional signal, then structured judgment—without deliberate conscious initiation, demonstrating the spontaneous ignition described in Section 5 of the main manuscript.
