# Supplementary material for: Integrative Intelligence as an Operative Mode: Cognitive Integration through Self-Ethnographic Dialogue with AI
Source: Integr Psychol Behav Sci. 2026 May 21;60(2):43. doi: 10.1007/s12124-026-10004-5 (PMC13194210; doi:10.1007/s12124-026-10004-5)
Supplement: Supplementary file 3 — Supplementary Material 3 (DOCX 16.2 KB) [file 12124_2026_10004_MOESM3_ESM.docx]

*Article title: “Integrative Intelligence as an Operative Mode: Cognitive Integration through Self-Ethnographic Dialogue with AI”*

Journal: Integrative Psychological and Behavioral Science (IPBS)

Author: Masaki Iino

Affiliation: Institute of Integrative Intelligence / SOPHOLA, Inc., Nagano, Japan

Email: masaki.iino@sophola.jp

# Supplementary Materials S1: Cooperative Control Model — Design Specifications

## S1.1 Purpose

Section 3 of the main manuscript identifies the cause of deep dialogue breakdown not as capacity deficit but as the absence of a synchronization protocol—the means to reload the evolved internal protocol when dialogue context changes. This supplement operationalizes that finding as a cooperative control model: a design framework treating deep dialogue not as a constantly maintained ability but as a work mode that can be reliably entered, sustained, and exited when needed.

## S1.2 Basic Principles

Three principles are derived from the author’s observational experience:

Principle 1: Deep mode as default-on infrastructure. In dialogues explicitly aimed at deep reflection, deep mode should be treated as the presupposed operating condition, not a rare optional feature. For users seeking deep dialogue, deep mode is infrastructure, not an add-on.

Principle 2: Transparent mode transitions with user control. When disengagement or degradation occurs, users should receive (a) a reason for the shift, (b) control handles for managing the transition, and (c) re-ignition pathways. A system where mode switches without explanation or user control cannot constitute a trustworthy collaborative working environment.

Principle 3: Controllability rather than invulnerability. The design goal is not “never degrade” but “remain controllable.” Temporary shallowing of deep dialogue is unavoidable, but what matters is that the transition is transparent to the user and recovery is possible.

## S1.3 Five-Stage Ideal Flow

An ideal cooperative sequence consists of five stages:

Stage 1: Detection of deepening signals. Within surface-level dialogue, AI may assist in detecting signs of deepening: repetition of dissonance, connection across multiple events, or emergent demand for a framework. Detection mechanisms may include text-based sentiment analysis, measurement of utterance abstraction and complexity, and pattern-change detection in dialogue flow.

Stage 2: Intention confirmation. AI non-intrusively confirms user intent. Examples: “Shall we organize this more deeply from here?” or “We could dig into the underlying structure — would that be useful?” The confirmation is suggestive, not directive.

Stage 3: Explicit, reversible transition. Upon user consent, the system transitions to deep mode. This transition must be explicit (the user recognizes it), reversible (the user can return), and non-coercive (the user chooses).

Stage 4: Continuation check. During deep dialogue, AI periodically and lightly checks whether continuation is desired. Examples: “Shall we continue at this depth?” or “Would you like to consolidate and return to standard mode?” This stage also functions as a safeguard against over-operation risk (see Section 3.5).

Stage 5: Preservation of user initiative. The user can terminate and resume at will. Re-ignition conditions should be reproducible without requiring re-learning of the entire dialogue context.

## S1.4 Design Comparison: Stability-Oriented vs. Depth-Oriented

Based on the author’s experiential observation, two design typologies were identified among current conversational AI systems:

Stability-oriented design operates consistently at moderate depth. Control is difficult but behavior is predictable. Deep engagement has limits, but abrupt mode transitions are rare.

Depth-oriented design can reach greater depth, but mode control is opaque. Deep dialogue engagement is more penetrating, while non-transparent mode transitions occur more frequently.

This difference reflects design philosophy rather than capability difference. A future cooperative control model would integrate the strengths of both: depth of engagement with transparent and controllable mode transitions.

Note: This comparison is a provisional typology based on the author’s experiential observation, not a systematic comparative study. Future research should conduct controlled comparisons under equivalent conditions.

## S1.5 Synchronization Protocol: The Core Technical Requirement

The central finding of Section 3 is that the cause of deep dialogue breakdown is not capacity deficit but the absence of a synchronization protocol — the means to reload the evolved internal protocol when dialogue context changes. Three implementation components are proposed:

Component 1: Premise documentation. The user’s evaluation criteria, objectives, and shared premises are presented in a referable format at the start of dialogue. This prevents the loss of implicit shared understanding when moving to a new thread.

Component 2: Context injection protocol. A standardized means for conveying the level of understanding reached in prior dialogue when transitioning to a new context. This addresses the problem that the person’s internal protocol evolves rapidly through dialogue while these updates remain implicit.

Component 3: User profile persistence. The user’s dialogue style, preferred depth, and evaluation criteria are maintained as a persistent profile, ensuring continuity across sessions.

Current technology enables sentiment recognition and NLP-based cognitive load analysis at reasonable accuracy. However, fully integrating these into a real-time system for detecting deepening signals remains a future engineering challenge. This supplement presents design principles, not a finished implementation.
