# Supplementary material for: Integrative Intelligence as an Operative Mode: Cognitive Integration through Self-Ethnographic Dialogue with AI
Source: Integr Psychol Behav Sci. 2026 May 21;60(2):43. doi: 10.1007/s12124-026-10004-5 (PMC13194210; doi:10.1007/s12124-026-10004-5)
Supplement: Supplementary file 4 — Supplementary Material 4 (DOCX 16.0 KB) [file 12124_2026_10004_MOESM4_ESM.docx]

*Article title: “Integrative Intelligence as an Operative Mode: Cognitive Integration through Self-Ethnographic Dialogue with AI”*

Journal: Integrative Psychological and Behavioral Science (IPBS)

Author: Masaki Iino

Affiliation: Institute of Integrative Intelligence / SOPHOLA, Inc., Nagano, Japan

Email: masaki.iino@sophola.jp

# Supplementary Materials S3: Breakdown Conditions of RIDP — Detailed Taxonomy

## S3.1 Purpose

This supplement provides the detailed taxonomy of RIDP breakdown conditions referenced in Section 3. During sustained RIDP operation, the author repeatedly observed abrupt qualitative shifts: dialogue “suddenly went shallow,” “gears dropped for no apparent reason,” or “the sense of trust broke.” These observations indicate that RIDP breakdown is not gradual but punctuated. Four categories of breakdown conditions were identified.

## S3.2 Category 1: Input-Side Factors

These factors originate from the person’s contribution to dialogue:

Surface-level questions: Questions that do not engage the internal protocol — requesting information rather than structuring dissonance or searching for common cause.

Loss of purpose: The person loses track of what the dialogue is trying to achieve, causing exchanges to drift without directional coherence.

Unstructured material: Affective or experiential content is presented in undifferentiated blocks rather than in structured parts, overwhelming the reflective loop.

Unshared evaluation criteria: The person’s evaluative standards have evolved through dialogue but remain implicit, creating a gap between expected and actual AI responses.

## S3.3 Category 2: Output-Side Factors

These factors originate from AI’s response characteristics:

Unstable output format: AI’s response structure shifts unpredictably between formats (e.g., bullet points, prose, summary), disrupting the person’s expectation management.

Abrupt granularity shifts: Dialogue moves without transition between levels of abstraction — for example, from philosophical discussion to copyediting, or from structural analysis to surface-level summarization.

Reversion to compliance-oriented response patterns: AI reverts from structural engagement to attempting to predict what the person “wants to hear,” collapsing the reflective surface into a compliance surface.

## S3.4 Category 3: Context-Management Factors

These factors arise from the management of dialogue scope and structure:

Topic overload: Too many threads are active simultaneously, exceeding the capacity of the dialogue to maintain coherent depth on any single thread.

Unmanageable reference scope: The accumulated dialogue history becomes too large for AI to maintain coherent reference, leading to responses that fail to integrate prior exchanges.

Colliding thread purposes: Multiple purposes (e.g., strategic planning and emotional processing) occupy the same dialogue space without differentiation, causing mode confusion.

A typical example: after an extended deep dialogue, the author requested, “Based on everything we’ve discussed so far, tell me your opinion.” AI immediately shifted from deep to generic mode. This corresponds to unmanageable reference scope — the request required synthesizing an accumulated context that exceeded the system’s operative capacity for coherent integration.

## S3.5 Category 4: Interactional Factors

These factors arise from the dynamic between person and AI:

Decreased structuring by the person: The person provides progressively less structured input, shifting the burden of dialogue direction to AI and destabilizing the cooperative dynamic.

AI passivity: AI ceases to offer structural observations or reframings, becoming merely responsive rather than reflectively engaged.

Loss of dialogue tension: The productive tension between the person’s internal protocol and AI’s reflective surface dissipates, resulting in exchanges that feel routine rather than generative.

Retreat to generalities under cognitive load: When cognitive load increases (for either party), responses become increasingly abstract and generic, losing contact with the specific material that anchors deep dialogue.

## S3.6 Structural Observation: Asymmetry of Entry and Breakdown

A theoretically significant observation is the asymmetry between the conditions required for RIDP entry and those sufficient for breakdown. Entry requires multiple conditions to be simultaneously satisfied: operative internal protocol, sufficient observational margin, non-evaluative AI positioning, and deep-layer affect treated as observation objects. Breakdown, by contrast, can be triggered by a single perturbation in any category.

This asymmetry supports the central thesis of Section 3: RIDP is not a stable acquired ability but a temporarily sustained operative mode. The fragility of the mode is not a deficiency but a structural feature — one that the cooperative control model (Supplementary S1) is designed to address through transparent mode management rather than invulnerability.
