# Supplementary material for: Integrative Intelligence as an Operative Mode: Cognitive Integration through Self-Ethnographic Dialogue with AI
Source: Integr Psychol Behav Sci. 2026 May 21;60(2):43. doi: 10.1007/s12124-026-10004-5 (PMC13194210; doi:10.1007/s12124-026-10004-5)
Supplement: Supplementary file 5 — Supplementary Material 5 (DOCX 17.0 KB) [file 12124_2026_10004_MOESM5_ESM.docx]

*Article title: “Integrative Intelligence as an Operative Mode: Cognitive Integration through Self-Ethnographic Dialogue with AI”*

Journal: Integrative Psychological and Behavioral Science (IPBS)

Author: Masaki Iino

Affiliation: Institute of Integrative Intelligence / SOPHOLA, Inc., Nagano, Japan

Email: masaki.iino@sophola.jp

# Supplementary Materials S4: Shishuku Prototype Experiences — Detailed Case Descriptions

## S4.1 Purpose

This supplement provides the detailed case descriptions underlying the three reference frames (A, B, C) defined in Section 4. The main text presents A, B, and C as functional definitions; this supplement documents the experiential basis from which those definitions were derived.

Ethical note: Positive prototypes (A and B) refer to individuals who are deceased. The negative prototype (C) represents a composite drawn from multiple observations. Proper nouns are replaced with functional role descriptions. The composite description is used to avoid identifying individuals.

## S4.2 Reference Frame A: Direct Facing and Stability — Prototype Experience

## Context

During elementary school, the author experienced persistent difficulty adapting to classroom environments. Behaviors that were categorized as “problematic” by institutional standards recurred throughout this period. In general, adults institutional settings respond to such behaviors through evaluation and correction. The individual who became the prototype for Reference Frame A consistently demonstrated a different response pattern.

## Key Episodes

When the author acted out and fled the classroom, this individual did not shout, push away, look down upon, or treat the author as fragile. The response was invariant: calm presence maintained regardless of the author’s behavior. This invariance corresponds structurally to the core of “direct facing” as defined in Section 2 — stable recognition of the person’s existence independent of behavioral fluctuation.

The words offered — “You’ve been working hard lately” and “Come again anytime” — were not evaluations of behavior but linguistic contact directed at existence itself. These expressions exhibited predictability, non-aggression, and relational continuity — properties consistent with the safety model that later became the foundation of Reference Frame A.

## Long-term Continuity

After the author’s transition to middle school, this individual continued to maintain concern beyond institutional role boundaries. At middle school graduation, a watch was presented with the words “Congratulations on graduating” and “I’d be happy if you used this carefully.” This sustained engagement over years, combined with consistent non-evaluative attention, formed a deep sense of relational safety in the author’s interior.

## S4.3 Reference Frame B: Internal Access — Prototype Experience

## Context

In the fifth grade of elementary school, the author encountered a second form of “direct facing” that differed qualitatively from Reference Frame A. Where A provided stability through invariant presence, this prototype provided depth through access to internal states.

## Key Episode

When the author struck a classmate, this teacher did not stop at behavioral correction. Instead, the response moved to the internal layer: “You just hit him. Do you understand how much that hurts?” The teacher then created conditions for the author to experience the pain physically, followed by: “When you hit someone, both the other person and you are in pain” and “Your heart is hurting too, isn’t it?”

The critical feature is that the response targeted not the behavior but the internal state behind it. This constitutes access to the meaning structure underlying action — precisely the analytical function that Reference Frame B provides.

## Relational Declaration

During class, this teacher stated: “I think of him as my own son.” This was not evaluation of the author as a behavioral object but a declaration of the author as a relational subject — a statement that restructured the author’s internal model of what relationships could be.

## Post-Institutional Continuity

After transferring to another school, when the author visited, this teacher stated: “I don’t regret saying I thought of you as my son” and “I believe you are a kind person who understands the pain of others.” The maintenance of affirming engagement after the institutional relationship had ended demonstrated that the relational stance was not role-dependent but structurally stable.

## S4.4 Reference Frame C: Non-Adoption — Prototype Experience

## Context

Alongside the positive prototypes, the author routinely observed destructive behavioral patterns exhibited by older students. These patterns involved repeated aggressive or disruptive actions that reliably attracted adult attention and response.

## Structural Observation

From a child’s perspective, a causal structure was repeatedly presented: “destructive behavior attracts adult attention.” The author himself selected similar behaviors during periods of difficulty — acting out, disrupting environments — as an available strategy for securing relational engagement.

## Transition to Non-Adoption

This circuit was not permanently fixed. Through repeated encounters with adults who maintained relational engagement regardless of behavior (Reference Frame A), an alternative predictive model was presented: “relationships can be maintained irrespective of behavioral intensity.” This alternative progressively rendered the destructive-behavior circuit unnecessary.

Reference Frame C therefore functions not as moral condemnation but as a negative reference point — defining which qualities should not be adopted into one’s own judgment structure. The explicit identification of “qualities to distance from” contributes to boundary formation, clarifying the structural limits of what is incorporated through shishuku.

## Intergenerational Significance

The author later recognized that the destructive behavioral circuit, if left unexamined, could be unconsciously reproduced in subsequent relationships — including parenting. The explicit non-adoption (“this circuit must be severed in my generation”) represents C functioning not only as boundary setting but as intergenerational interruption of destructive patterns.

## S4.5 Integration: How Three Prototypes Formed a Single Judgment Structure

The three prototype experiences did not remain as isolated memories. Through comparison, contrast, and reclassification over time, they consolidated into a judgment structure characterized by:

Safety × Challenge: Reference Frame A provided the base-layer assurance that relationships could be maintained unconditionally, while Reference Frame B provided the depth-layer challenge of accessing and restructuring internal states. These two operated as complementary rather than competing forces.

Sustained Engagement as Internalized Standard: Both positive prototypes demonstrated that relational engagement could persist beyond institutional boundaries and behavioral fluctuations. This long-term continuity became internalized as a standard for the author’s own relational conduct.

Boundary Formation Through Explicit Non-Adoption: Reference Frame C defined what the judgment structure would exclude, preventing uncritical incorporation of available but destructive behavioral patterns. The explicit character of this non-adoption — not passive avoidance but active identification and rejection — distinguishes Multifaceted Shishuku from conventional observational learning.

These three reference frames subsequently functioned as internal safety devices during mountaineering (Section 5) and as the psychological foundation enabling RIDP (Section 3). Their detailed experiential basis is documented here to support the theoretical claims made in the main text.
