# Supplementary material for: Integrative Intelligence as an Operative Mode: Cognitive Integration through Self-Ethnographic Dialogue with AI
Source: Integr Psychol Behav Sci. 2026 May 21;60(2):43. doi: 10.1007/s12124-026-10004-5 (PMC13194210; doi:10.1007/s12124-026-10004-5)
Supplement: Supplementary file 6 — Supplementary Material 6 (DOCX 24.3 KB) [file 12124_2026_10004_MOESM6_ESM.docx]

*Article title: "Integrative Intelligence as an Operative Mode: Cognitive Integration through Self-Ethnographic Dialogue with AI"*

Journal: Integrative Psychological and Behavioral Science (IPBS)

Author: Masaki Iino

Affiliation: Institute of Integrative Intelligence / SOPHOLA, Inc., Nagano, Japan

Email: masaki.iino@sophola.jp

# Supplementary Materials S6: Verbatim Records and Physiological Data — Three Mountaineering Cases

This supplement provides the detailed verbatim records, physiological data, and phase-by-phase narrative descriptions for the three mountaineering case studies summarized in Sections 5.4–5.6 of the main manuscript. Records documented during or immediately after action are treated as primary data; retrospective interpretations are analytically distinguished and indicated as such. All verbatim records are translated from Japanese field notes. Physiological data is drawn from Garmin wearable device logs.

# S6.1 Case Study 1: Mt. Utsugi — Introspective Mode

Mt. Utsugi (2,864m, southern Japanese Alps) was climbed solo in early winter via the Ikeyama Ridge: 12-hour action with 2,000m+ elevation gain under darkness, snow/ice, and progressive fatigue. This case was selected because the transitional season—where neither full winter equipment nor summer-style judgment can be stably relied upon—creates persistent conditions that cannot be subsumed under existing manuals or established protocols.

### Serial Circuit Establishment (04:16–05:27)

In the dark approach, early morning darkness around 4 AM, low temperature, limited visibility, and absence of others overlapped, establishing an isolated judgment environment simultaneously with action start. "Primal fear" was recorded in response to animal sounds and grass swaying in the wind, but judgment was not delegated externally; criteria such as "walk slowly" and "retreat if it becomes impossible" were generated internally. No circuit for referring to external evaluation or advice existed from the outset—judgment converged on the question: "What do I do now, in this situation?"

During this dark walking, a childhood experience of a "world without light" was recalled, and the memory of continuing to walk with anxiety not knowing which direction to go overlapped with present bodily sensation. When looking back, the lights of Komagane city were recognized as "light," and the gradual brightening of the eastern sky was recorded as "This is hope." Darkness as a natural condition did not explain whether progress was possible; it functioned as an existence that returned only results (Condition 3).

These internal dialogues, based on primary records from during or immediately after action, can be organized as meaning generation synchronized with the environmental stimulus of darkness and bodily state. Rather than negating fear, they fulfilled the function of internally reconstructing "conditions for continuing to advance even in darkness"—meaning-making functioning not as emotional processing but as an internal support structure for sustaining judgment. In this phase, fear arose first as bodily sensation, then meaning-making was generated, and as a result, judgment stabilized: a serial circuit (Condition 4) of body → emotion → meaning → judgment was clearly established.

The judgment criteria arising at this moment—"walk slowly," "retreat if it becomes impossible"—can be read as a phase where reference frames derived from Multifaceted Shishuku figures (Section 4: A, direct facing and stability; C, retreat judgment as boundary setting) held the deep-layer fluctuation originating from the body and grounded judgment in reality conditions. Rather than denying fear, safety was secured by always leaving retreat possibility—a sign that internal others were functioning as safety devices under the constraint configuration. This pattern of maintaining retreat possibility resonates with Crust et al.'s (2016) finding that experienced mountaineers recognized "the thin line between persevering and over-stretching," though the present study specifies this not as a personality trait but as a structural feature of judgment operating under the four conditions.

### Deepening Under Accumulated Constraint (08:20–11:50)

From Mayoi Ridge onward, complex environmental factors—snow, ice, rocky ridges, narrow ridges, and rapid cooling from wind—were concentrated. Under this configuration, environmental changes occurred continuously, and judgment did not complete with each instance but accumulated in a form that physically constrained subsequent options.

During this process, judgment strategies such as "focus on each step at my feet" and "don't look at distant goals" were recorded. These indicate that continuous judgment responding to environmental change and bodily load (Condition 2) crystallized into concrete action policies. Additionally, memories of sales activities during a business crisis period and bodily sensations formed during past difficult climbs (Kuroto Ridge) were recalled. These recollections were not retrospective reflection; they fulfilled the role of connecting strategies such as "don't look far, advance step by step" and "divide and process the load" to present action as judgment resources. Here too, judgment was generated not thought-first but originating from bodily state and environmental stimulus.

Garmin data showed heart rate sustained in the 130–150 bpm range for extended periods, with high-frequency repetition of stops and restarts. Despite bodily margin not being large, the Metacognitive Camera (Section 2) continued to function as observational attitude, and the three-person conference was maintained without collapse. As physical limits approached, judgment converged on reference frames of "step by step" and "leaving retreat possibility" rather than abstract optimization—the internal protocol continuing to operate as minimal structure.

This indicates a boundary condition where, even under sustained high load, the serial circuit prevented judgment from deviating into either abstract planning or emotional runaway. At the point of reaching the summit, action did not remain a temporary achievement but continued into the next descent judgment. Completion was not made into a goal; summit attainment was incorporated as one phase of the judgment process, not as an endpoint requiring celebration or evaluation.

### Continuity Under Depletion (13:19–16:31)

In the late descent, depletion of leg strength and stamina was recorded. In this phase, physical limits clearly came to the foreground, but judgment did not become abstract; it directly connected to concrete actions such as "careful, slow descent" and "conscious practice of descent techniques." Here too, judgment was generated originating from bodily state, and fear and haste did not precede thought. Because the serial circuit (Condition 4) was maintained, judgment did not diverge from reality conditions, and action continued without breakdown.

A critical observation in this phase concerns the role of the Metacognitive Camera. As bodily load increases, the MC may incline toward "control"—evaluating, optimizing, attempting to direct rather than observe (see Section 2). In this case, observation and retention rather than evaluation and optimization remained predominant, which contributed to maintaining safety. The natural environment continued to return only results without evaluation; the isolated judgment environment was maintained to the end, and judgment reached completion without being pulled back to external criteria.

# S6.2 Case Study 2: Mt. Amari — Relational Mode

Mt. Amari (~2,000m) and Mt. Sentōboshi were traversed with the author's 6-year-old son in autumn: 5-hour action with moderate load. Descriptions of the companion serve to describe judgment processes within relationships and condition structures, not for developmental evaluation.

### Isolation Despite Accompaniment (08:18–08:43)

Despite accompaniment, an isolated judgment environment was established. Isolation here is not physical solo action but the structure where judgment responsibility is concentrated in one person regarding route, rest, and continuation. When the child responded "I won't go because I'll get tired" to the suggestion of continuing to Mt. Oku-Amari, the author changed the initially planned route and aimed for Mt. Sentōboshi—continuous judgment incorporating the companion's state as environmental information, not external evaluation.

### Relational Extension of the Serial Circuit (08:43–09:18)

In the first half of the traverse, several steep climbs continued, and the child's fatigue gradually manifested. A short break was inserted, and recovery was observed through spreading out snacks. In this rest phase, within the author's interior, the memory of "feeling mountaineering like a picnic when I was a child" was recalled. This can be organized as meaning generation that arose to readjust present action judgment rather than retrospective sentiment.

That is, the child's fatigue (bodily state), recovery and enjoyment through snacks (emotional response), and the author's own childhood memory (meaning) were connected serially, and as a result, judgments such as "take a longer break" and "redesign the itinerary from the child's perspective" were generated. Here, the serial circuit (Condition 4) of body → emotion → meaning → judgment operated originating from relationship. The circuit's input was not the author's own body but the other's bodily state—a relational extension of the same structural mechanism observed in Case 1.

### Intergenerational Vertical Axis Integration (09:18–10:27)

After crossing a considerable steep climb, rest was taken at a large rock before the Gosho-yama junction. The child was visibly exhausted—complaining, dragging his feet—but he had walked through. The distance and cumulative elevation gain were equivalent to roughly twice the local village mountains. Sitting on that rock, looking at this small body that had not given up, the author found himself speaking words he had not planned: affirming the child's effort, telling him that what he had just done was something real.

At the same moment, a memory surfaced: his own childhood, and the rare occasions when effort had been seen and praised—episodes that had remained disproportionately vivid in autobiographical memory decades later. The structural observation here is that the author's vertical axis (Section 2) activated not through deliberate recall but through the child's bodily state as trigger: past self (effort not witnessed) and future self (what this child may carry forward) were simultaneously present in the judgment generating the affirmation. This is not sentiment but temporal integration constrained to activate by relational configuration.

What was observed here is not mere encouragement but a structure where three temporal perspectives were simultaneously active: the past (memory of oneself not being seen), the present (the child's body completing the walk before one's eyes), and the future (the possibility that the experience of "effort being recognized" would become internalized for the child). The speech was not monologue; it was a process where intergenerational time was integrated through words directed at another. The author's own meaning structure was reorganized in the same moment that conditions were created for the child to form the understanding that "effort is seen and has value."

Unlike the elaborative reminiscing described in the parent-child memory literature (Fivush, 2019; Fivush et al., 2004), where narration occurs after events in conversational settings, the meaning generation observed here was embedded in ongoing action under environmental constraint—arising spontaneously as the child's bodily state activated the parent's temporal integration circuit. The parent's internal protocol did not merely scaffold the child's memory; it simultaneously reorganized the parent's own judgment structure.

### Affirmation as Behavioral Fixation (11:22–13:24)

After rest and a meal at the summit of Mt. Sentōboshi, the child recovered both physically and emotionally. After beginning the descent, action stabilized, and the child repeatedly said, "Thank you for bringing me to a fun mountain"—a verbatim record indicating that the preceding positive experience had been internalized. Furthermore, the child spontaneously proposed a detour to Mt. Oku-Amari—a sign that the affirmation had re-emerged as action motivation. The author readjusted the itinerary after confirming safety conditions. Here too, judgment was not plan-adherent but generated as continuous judgment incorporating the companion's state (Condition 2).

### Perceptual Redefinition Through Encounter

Near Mt. Amari summit, they encountered a volunteer performing grass-cutting. The child spoke "Ah, a dragonfly!" and instantly noticed a dragonfly ornament attached to the volunteer's hat—an object the author had not noticed at all. With this perceptual asymmetry as trigger, within the author's interior, observation occurred that the child's characteristics were reinterpreted as valuable perceptual ability in a context different from everyday life. What happened here is not evaluation of the companion but a process where meaning-making on the horizontal axis (roles and relationships) was updated, and the author's attention allocation and value weighting were rearranged.

# S6.3 Case Study 3: Mt. Togakushi-Nishi — Immediate Mode

Mt. Togakushi-Nishi is a peak in the Togakushi mountain range (northern Nagano) where general hiking trails and technical elements are mixed: broken-line sections (unmarked trail segments requiring route-finding judgment), continuous vertical chains, ladders, and traverses with no bypass routes, where judgment errors could lead to serious consequences. Total action time was 12.5 hours; Garmin data showed average heart rate 143 bpm, maximum 175 bpm, with 59% of action time in zone Z4 (high-intensity aerobic effort, approximately 80–90% of maximum heart rate). This case was selected to observe how integrative operation is constrained in high-risk environments where judgment delay itself becomes risk—judgment as immediate output during execution, not understanding through reflection.

### Constraint Accumulation

The action involved 12.5 hours of continuous movement with approximately 1,500m cumulative elevation gain. Multiple constraints accumulated progressively beyond mere physical fatigue: route uncertainty from the broken-line section after the pasture, high technical demands from vertical chains, ladders, and lateral traverses with no bypass routes, strong sunlight and high-temperature environment, and rest being physically obstructed by countless small insects and monkey intimidation—meaning that even when the body demanded recovery, the environment refused to provide conditions for it. The mobile phone was dropped off a cliff in the broken-line section and recovered, and subsequently in the core section, a trekking pole was lost. "This made the rest of the mountain trip considerably harder" was recorded—constraint accumulation verbalized as situation assessment, the loss of a compensatory tool compounding the already narrowing margin.

Normally, high physiological load works to lower cognitive function. However, in this case, phases where judgment appeared to "sharpen" were observed. This sharpening was not ability elevation but a result where strong survival demand eliminated many unnecessary horizontal axes (social roles, distractions, circuits of external evaluation) and concentrated judgment resources on the deep layer (bodily constraints) and the vertical axis (immediate causality: this one move directly impacts future action possibility).

### Embodied Protocol Under High-Risk Terrain (P1 Onward)

In the section from P1 onward, vertical chains and slippery steep slopes continued. "Absolutely no rest" and "considerably exhausted" were recorded as bodily responses—not reflective assessments but real-time registrations of a body approaching its limit while continuing to move. In these sections, judgment delay or stopping itself could lead to falls—an environment where integrative operation was constrained to immediate form.

The internal protocol (three-person conference and Metacognitive Camera described in Section 2) did not operate as deliberative dialogue but as what this study terms "embodied protocol": synchronized with bodily movement and tightly coupled with action, outputting judgment without separation from physical execution. Each chain, each lateral traverse required immediate judgment that maintained multiple information layers—bodily state, emotional response, environmental risk, and action possibility—simultaneously without fragmentation.

### Judgment Conversion at Critical Threshold

The decisive event occurred at Happō-nirami where all 3.6L of drinking water was consumed. At this point, two verbatim records capture the escalating situation assessment:

*"In this condition, the Ant's Tower Traverse..."*

The Ant's Tower Traverse is a knife-edge ridge with sheer drops on both sides, requiring sustained technical movement with no margin for hesitation—precisely the kind of terrain that demands maximum physical and cognitive resources. The ellipsis in the verbatim record itself captures the moment where language fails to complete the thought, and bodily dread fills the gap.

*"Barely at the line where if things go wrong, I'd have to request rescue due to becoming unable to act."*

Here, bodily constraints (water depletion, cumulative fatigue), emotional response (urgency, recognition of proximity to limit), and environmental risk assessment (high failure cost of the remaining technical terrain) arose serially, and as a result, an explicit judgment conversion was generated: "anyway, take it slowly and safely descend without overexertion."

On verbatim data, this judgment conversion arose with bodily constraints and risk assessment maintained simultaneously—it emerged as judgment outputted immediately without fragmenting multiple information layers. In this environment, judgment delay itself becomes risk, so internal dialogue operated as an immediate judgment generation process rather than extended deliberation. What is characteristic is that the conversion maintained retreat possibility under extreme constraint: it was not a failure or retreat in the evaluative sense but a structural response to constraint accumulation that preserved survival possibility.

### Recovery and Continuation

"I felt like I was truly coming back to life" was recorded at the phase of reaching Togakushi Shrine inner sanctuary and hydrating—a moment where bodily state recovery was clearly recognized subjectively. Subsequently, no judgment breakdown or accidents were recorded in the descent process, and the traverse was completed leading to safe descent. This continuation was established not as reckless advance under high-risk conditions but as convergence of action including appropriate judgment conversion. The judgment conversion at the critical point functioned as protection against breakdown, suggesting that integrative operation under extreme constraint can sustain itself through structural response rather than willpower alone.

# Note on Data Sources

All verbatim records presented in this supplement were documented during or immediately after action and are treated as primary data. Retrospective interpretations are analytically distinguished and used only as supplementary material. Physiological data (heart rate, exertion zones) is drawn from Garmin wearable device logs. Mountaineering activity records (route, timing, elevation) are from Yamareco activity logs. Proper nouns identifying specific locations beyond those named in the main text are retained here as they appear in the original field records.

**References**

Fivush, R. (2019). Sociocultural developmental approaches to autobiographical memory. *Applied Cognitive Psychology*, *33*(4), 489–97. https://doi.org/10.1002/acp.3512

Fivush, R., Bohanek, J., Robertson, R., & Duke, M. (2004). Family narratives and the development of children’s emotional well-being. In M. W. Pratt & B. H. Fiese (Eds.), *Family stories and the life course: Across time and generations* (pp. 55–76). Lawrence Erlbaum Associates.
